# The Effect of Visfatin on the Functioning of the Porcine Pituitary Gland: An In Vitro Study

**DOI:** 10.3390/cells12242835

**Published:** 2023-12-14

**Authors:** Karolina Szymanska, Edyta Rytelewska, Ewa Zaobidna, Marta Kiezun, Marlena Gudelska, Grzegorz Kopij, Kamil Dobrzyn, Ewa Mlyczynska, Patrycja Kurowska, Barbara Kaminska, Anna Nynca, Nina Smolinska, Agnieszka Rak, Tadeusz Kaminski

**Affiliations:** 1 Department of Animal Anatomy and Physiology, Faculty of Biology and Biotechnology, University of Warmia and Mazury in Olsztyn, 10-719 Olsztyn, Poland; k.szymanska@uwm.edu.pl (K.S.); edyta.rytelewska@uwm.edu.pl (E.R.); marta.kiezun@uwm.edu.pl (M.K.); grzegorz.kopij@uwm.edu.pl (G.K.); barbara.kaminska@uwm.edu.pl (B.K.); anna.nynca@uwm.edu.pl (A.N.); nina.smolinska@uwm.edu.pl (N.S.); 2 Department of Biochemistry, Faculty of Biology and Biotechnology, University of Warmia and Mazury in Olsztyn, 10-719 Olsztyn, Poland; ewa.zaobidna@uwm.edu.pl; 3 Department of Human Histology and Embryology, School of Medicine, Collegium Medicum, University of Warmia and Mazury in Olsztyn, 10-082 Olsztyn, Poland; marlena.gudelska@uwm.edu.pl; 4 Department of Zoology, Faculty of Biology and Biotechnology, University of Warmia and Mazury in Olsztyn, 10-719 Olsztyn, Poland; kamil.dobrzyn@uwm.edu.pl; 5 Laboratory of Physiology and Toxicology of Reproduction, Institute of Zoology and Biomedical Research, Jagiellonian University in Krakow, 30-387 Krakow, Poland; ewa.mlyczynska@uj.edu.pl (E.M.); patrycja.kurowska@uj.edu.pl (P.K.); agnieszka.rak@uj.edu.pl (A.R.); 6 Doctoral School of Exact and Natural Sciences, Jagiellonian University in Krakow, 30-348 Krakow, Poland

**Keywords:** visfatin, FK866, LH, FSH, cell proliferation, apoptosis, signaling pathways, estrous cycle

## Abstract

Visfatin (VIS), also known as nicotinamide phosphoribosyltransferase (NAMPT), is the rate-limiting enzyme in the biosynthesis of nicotinamide adenine dinucleotide (NAD+). Recently, VIS has been also recognized as an adipokine. Our previous study revealed that VIS is produced in the anterior and posterior lobes of the porcine pituitary. Moreover, the expression and secretion of VIS are dependent on the phase of the estrous cycle and/or the stage of early pregnancy. Based on this, we hypothesized that VIS may regulate porcine pituitary function. This study was conducted on anterior pituitary (AP) glands harvested from pigs during specific phases of the estrous cycle. We have shown the modulatory effect of VIS in vitro on LH and FSH secretion by porcine AP cells (determined by ELISA). VIS was also found to stimulate cell proliferation (determined by Alamar Blue) without affecting apoptosis in these cells (determined using flow cytometry technique). Moreover, it was indicated that VIS may act in porcine AP cells through the INSR, AKT/PI3K, MAPK/ERK1/2, and AMPK signaling pathways (determined by ELISA or Western Blot). This observation was further supported by the finding that simultaneous treatment of cells with VIS and inhibitors of these pathways abolished the observed VIS impact on LH and FSH secretion (determined by ELISA). In addition, our research indicated that VIS affected the mentioned processes in a manner that was dependent on the dose of VIS and/or the phase of the estrous cycle. Thus, these findings suggest that VIS may regulate the functioning of the porcine pituitary gland during the estrous cycle.

## 1. Introduction

The pituitary gland plays a crucial role in controlling essential biological processes in humans and animals, including growth, metabolism, and reproduction [1]. As a result, disruptions in pituitary functioning can lead to various hormonal and metabolic disorders [2]. The activity of the pituitary gland is mainly regulated by feedback loop mechanisms, which involve numerous hormones [1]. However, it can also be influenced by other biological triggers that modulate the hormonal and metabolic states of organisms. An increasing number of studies suggest that adipokines, a group of factors primarily produced and secreted by the adipose tissue, may have a significant impact on the functioning of the pituitary gland [3]. One of these adipokines is visfatin (VIS), also known as nicotinamide phosphoribosyltransferase (NAMPT) [4].

VIS was initially identified as a pre-B cell colony-enhancing factor; however, subsequent research has shown that it may play an enzymatic role in the production of nicotinamide adenine dinucleotide (NAD+) [5]. VIS exists in two isoforms: intracellular (iNAMPT) and extracellular (eNAMPT) [6]. iNAMPT plays a regulatory role in NAD+ biosynthesis and can affect many NAD+-dependent proteins such as sirtuins, poly(ADP-ribose) polymerases, mono(ADP-ribosyl) transferases, and CD38/157. Conversely, eNAMPT is associated with hormone-like signaling pathways and activates intracellular signaling cascades, including protein kinase B/phosphatidylinositol 3-kinase (AKT/PI3K), mitogen-activated protein kinase/extracellular signal-regulated kinase (MAPK/ERK), and adenosine monophosphate-activated protein kinase (AMPK) [7,8]. It is important to note that although the actions of VIS were initially thought to be mediated by the insulin receptor (INSR) [9], C-C chemokine receptor type 5 [10], and toll-like receptor 4 [11], to date, no specific receptor for this adipokine has been officially recognized.

So far, VIS gene expression has been found in sheep in a part of the anterior pituitary (AP) lobe known as the pars tuberalis, which is thought to regulate seasonal physiology by decoding changes in nocturnal melatonin secretion [12]. A subsequent study has shown the gene and protein expression of VIS in a mouse gonadotroph cell line, capable of producing gonadotropins [13]. This study also demonstrated an inhibitory effect of VIS on LH secretion by these cells. Further, in our previous research, it has been found that VIS is also produced in both the anterior and posterior pituitary lobes of the domestic pig. Additionally, VIS expression and secretion were shown to be dependent on the phase of the estrous cycle and/or the stage of early pregnancy [14]. Furthermore, VIS secretion by porcine anterior pituitary cells (APc) was found to be influenced by gonadotropin-releasing hormone (GnRH), luteinizing hormone (LH), follicle-stimulating hormone (FSH), and insulin (INS), also in a phase-dependent manner [14]. However, the precise role of VIS in the regulation of pituitary function, especially in relation to female reproduction, remains unknown. This formed the basis of the research hypothesis that VIS may regulate the functioning of the porcine pituitary during the estrous cycle, which prompted us to conduct this study.

The primary role of the pituitary gland is the production and secretion of gonadotropins, including LH and FSH, that are essential for the development and function of the female gonads. Therefore, the primary objective of this study was to investigate the in vitro effect of VIS on the secretion of LH and FSH by porcine APc during the estrous cycle (Experiment number 1—Expt. No. 1). Furthermore, the functioning of the pituitary depends on proliferation and apoptosis, two dynamic and continuous processes that are essential for maintaining an optimal number and variety of cells. As these processes may influence the secretory functions of the pituitary, this study also aimed to evaluate the in vitro effect of VIS on the proliferation of cells within the porcine AP gland (Experiment number 2—Expt. No. 2), as well as on the apoptosis process in these cells (Experiment number 3—Expt. No. 3). Importantly, to comprehend the role of VIS in the processes of gonadotropins secretion, cell proliferation, and apoptosis, in all three experiments, porcine pituitary cells were cultured in the presence and absence of VIS or/and FK866—a specific and selective VIS inhibitor that also blocks its enzymatic activity. To elucidate the exact mechanism of VIS action in the porcine AP gland, we also investigated the in vitro effect of VIS on the activation of the INSR, AKT/PI3K, MAPK/ERK1/2, and AMPK signaling pathways during the mid-luteal phase of the estrous cycle—the phase in which the activity of the corpus luteum is the highest throughout the cycle: days 10–12 (Experiment number 4—Expt. No. 4). Finally, we investigated the in vitro effect of VIS on LH and FSH secretion by porcine APc in the presence of highly selective inhibitors of the INSR, AKT/PI3K, MAPK/ERK1/2, and AMPK signaling pathways (Experiment number 5—Expt. No. 5).

## 2. Materials and Methods

### 2.1. Experimental Animals and Tissue Collection

This study was conducted on AP glands harvested from pigs destined for commercial slaughter and meat processing. Collection of the material and its use for research were in accordance with the Act of the 15 January 2015 (Journal of Laws 2015, item No 266) on the Protection of Animals Used for Scientific or Education Purposes and Directive 2010/63/EU of the European Parliament and the Council of the 22 September 2010 on the Protection of Animals Used for Scientific Purposes. The experimental group consisted of mature cross-breed gilts (Large White × Polish Landrace) aged 7–8 months, and weighing 130–150 kg. The animals’ diet was balanced according to the current Polish nutritional standards for pig breeding. The AP glands were collected from gilts (*n* = 5 per group, 20 in total) during specific phases of the estrous cycle: early luteal phase (luteinization phase; days 2–3), mid-luteal phase (the phase in which the activity of the corpus luteum is the highest throughout the cycle; days 10–12), late luteal phase (luteolysis phase, days 14–16) and the follicular phase (days 17–19). The phase of the estrous cycle was determined by the morphology of the ovaries [15]. The pituitary glands were harvested immediately after slaughter, placed in ice-cold Dulbecco’s phosphate-buffered saline (D-PBS; pH = 7.4) with an antibiotic mixture (Antibiotic-Antimycotic Solution; Merck, Darmstadt, Germany), and then transported on ice to the laboratory within 1–1.5 h. The purity of the anterior lobe collection was confirmed by microscopic examination (as attached in Supplementary Figure S1).

### 2.2. Cell Isolation and In Vitro Cultures

The procedure of APc isolation was performed as described by Szymanska et al. [14]. In brief, the AP lobes were cut into small pieces. Then, isolation of the cells was performed through digestion of the pituitary lobes with 0.2% collagenase (Merck, Darmstadt, Germany) at 37 °C for 30 min. The dispersed APc were transferred into 15 mL tubes containing Dulbecco’s medium and centrifuged three times at 800× *g*, at room temperature for 10 min. From the second digestion cycle, the cells were treated with a solution containing collagenase 0.2% and pancreatin 0.25% (Merck, Darmstadt, Germany) for 10 min each, until the whole tissue was dispersed. After each digestion cycle, the dispersed cells were collected and washed as described above. After isolation, APc viability was assessed using a dye exclusion test with a 0.4% trypan blue solution (Merck, Darmstadt, Germany). The average viability of each cell isolation was greater than 90%. These cells were then used to establish in vitro cultures, suitably designed for each experiment. For the enzyme-linked immunosorbent assay (ELISA; Expt. Nos. 1 and 5) and flow cytometry technique (Expt. No. 3), APc were seeded on 24-well plates at a concentration of 2.5 × 10^5^ cells/well in 1 mL of medium. For the Alamar Blue assay (Expt. No. 2), APc were seeded on 96-well plates at a concentration of 1 × 10^5^ cells/well in 100 μL of the medium. In turn, for the Western Blot analysis (Expt. No. 4), APc were seeded on 6-well plates at a concentration of 1.5 × 10^6^ cells/well in 2 mL of medium. The APc were preincubated in McCoy’s-5A medium (Merck, Darmstadt, Germany) supplemented with 10% horse serum (Merck, Darmstadt, Germany), 2.5% fetal calf serum (Merck, Darmstadt, Germany), and a mixture of antibiotics (Antibiotic-Antimycotic Solution; Merck, Darmstadt, Germany) for 48 h at 37 °C.

In a humidified atmosphere of 5% CO_2_ and 95% air, fresh serum-free medium (other. components unchanged) was then added and preincubation was continued for a further 24 h (37 °C, 5% CO_2_, and 95% air). After 72 h of preincubation, the medium was removed and replaced with fresh serum-free medium, and the cells were cultured for the specified time with or without treatments—the experimental setup is presented in Table 1. The entire experimental setup was performed in duplicate. The cells incubated with serum-free medium, without any treatment, served as controls. The doses of VIS—1, 10, and 100 ng/mL—represented subphysiological, physiological, and supraphysiological concentrations of VIS, respectively. These doses were chosen on the basis of our recent study [16] in which we determined the plasma concentration of VIS in pigs.

The dose of the inhibitor FK866 (10 nM; Merck, Darmstadt, Germany) was chosen on the basis of Reverchon et al. [17]. The doses of GnRH (100 ng/mL; Merck, Darmstadt, Germany) and INS (10 ng/mL; Merck, Darmstadt, Germany) were chosen according to the studies of Bogacka et al. [18] and Gavin et al. [19], respectively. In turn, the doses of S961 (1 μM; an inhibitor of the INSR signaling pathway; Merck, Darmstadt, Germany), LY294002 (20 μM, an inhibitor of the AKT/PI3K signaling pathway; Merck, Darmstadt, Germany), U0126 (10 μM, an inhibitor of the MAPK/ERK1/2 signaling pathway; Merck, Darmstadt, Germany), and Dorsomorphin/Compound C (10 μM, an inhibitor of the AMPK signaling pathway; Merck, Darmstadt, Germany) were selected according to the studies of Elliott et al. [20], Zhao et al. [21], and Reverchon et al. [17]. After incubation, the culture media for the ELISA were collected and centrifuged at 1000× *g* for 10 min at 4 °C. The supernatants (media samples) were stored at −20 °C for further analysis. For the Western Blot technique, the culture media were removed, and the cells were washed twice with PBS and lysed with T-PER Tissue Protein Extraction Reagent (Cat. No. 78510; Thermo Fisher Scientific, Waltham, MA, USA), according to manufacturer’s instructions. The protein lysates were centrifuged at 10,000× *g* for 5 min at 4 °C and then stored at −80 °C for further analysis. In turn, the Alamar Blue assay and flow cytometry technique were performed immediately after completion of the in vitro cell culture, and the details of these methods are described in the respective subsections.

### 2.3. Gonadotropins’ Secretion—ELISA (Expt. Nos. 1 and 5)

The concentrations of gonadotropins in the culture media were determined using commercially available ELISA kits for porcine proteins, according to the manufacturers’ protocols. Details of the used ELISA kits, including the catalog number, name of the supplier, type of ELISA, sensitivity of the assay, and detection range, are given in Table 2. According to the manufacturers, no significant cross-reactivity or interference between target proteins and their analogs was observed. A preliminary test was performed prior to the main analysis to determine the optimal dilution of the culture media to ensure the most effective detection range for the kit. As a result, media samples were two-fold diluted for the LH ELISA test and ten-fold for the FSH ELISA test. Absorbance values were measured at 450 nm using the Infinite M200 Pro reader with Tecan i-control™ 1.8 software (Tecan, Mannedorf, Switzerland). All media samples were run in duplicate. The intra- and inter-assay coefficients of variation for the assays are shown in Table 2. The concentrations of the target proteins in the samples were interpolated from the standard curves, and the fit of the standard curves was confirmed by the coefficient of determination (R^2^), shown in Table 2.

### 2.4. Cell Proliferation—Alamar Blue Assay (Expt. No. 2)

The proliferation of APc was assessed using an Alamar Blue^®^ (AB; Thermo Fisher Scientific, Waltham, MA, USA) assay, according to the manufacturer’s instructions. Immediately after completion of the in vitro culture, the absorbance for each well on the plates was measured at two wavelengths, 570 nm (A570) and 600 nm (A600), using an Infinite M200 Pro spectrophotometer (Tecan, Mannedorf, Switzerland). To calculate the percentage difference in Alamar Blue^®^ dye reduction by cells incubated in the presence of specific treatments, we compared the ratio (A570-A600)/(A600) of background corrected absorbance values for media samples from the treatment groups with the same ratio for media samples from the control group. The obtained results represented the metabolic activity of the cells and indicated the resulting tendency for cell proliferation. Values close to 100% were interpreted as indicating no change in cell proliferation, values above 100% were considered indicative of stimulated cell proliferation, and values below 100% were considered indicative of inhibited cell proliferation.

### 2.5. Cell Apoptosis—Flow Cytometry Technique (Expt. No. 3)

The process of apoptosis of APc was determined by dual-color analysis using the flow cytometry technique. The Swine Annexin V-Fluorescein Apoptosis Assay Kit (ImmunoChemistry Technologies, Davis, CA, USA) was used to examine this process, according to the manufacturer’s instructions. The green-fluorescent Annexin V was used to label cells in the early phase of apoptosis, while the red-fluorescent Propidium Iodide (PI) stain was used to detect cells in the late stage of apoptosis as well as dead cells. The positive control group for apoptosis consisted of cells treated with camptothecin (Merck, Darmstadt, Germany) at a dose of 4 µg/mL, as recommended in the protocol of the Apoptosis Assay Kit. For the analysis, the media were removed immediately after the end of the in vitro culture and the cells were dissociated with 0.05% trypsin-EDTA (Merck, Darmstadt, Germany). The cells were then washed twice with PBS and centrifuged at 1000× *g* for 5 min at room temperature (RT). APc were then suspended at a concentration of 1 × 10^6^ in 0.4 mL of a calcium-based binding buffer. Flow cytometric measurements were performed using a FACSCelesta™ flow cytometer (BD Biosciences, Franklin Lakes, NJ, USA). Data were acquired and analyzed using FACSDiva 9.0 software (BD Biosciences, Franklin Lakes, NJ, USA). Both data acquisition and result analysis were performed on 20,000 events.

### 2.6. Activation of Signaling Pathways—ELISA and Western Blot Analysis (Expt. No. 4)

The activation of the INSR pathway was assessed by quantifying the concentrations of total INSR (total-INSR) and its phosphorylated form (phospho-INSR) in protein extracts using commercially available ELISA kits designed for porcine proteins (details of the used ELISA kits, including the supplier name, catalog number, and ELISA type, are given in Table 2). The assay was performed according to the manufacturer’s protocols. Prior to the main analysis, a preliminary test was performed to determine the appropriate amount of protein extracts required to ensure the optimal detection range of the kit. As a result, 160 µg of protein per well was used for each assay. Absorbance values were measured at 450 nm using the Infinite M200 Pro reader operated with Tecan i-control™ 1.8 software (Tecan, Mannedorf, Switzerland). All samples were assayed in duplicate. They were all evaluated on a single plate and the intra-assay coefficients of variation for these assays are shown in Table 2. The concentrations of the target proteins in the samples were interpolated from the standard curves, and the fit of the standard curves was confirmed by the coefficient of determination (R^2^), shown in Table 2. The ratio between the concentrations of phospho-INSR and total-INSR for each sample from the same extraction was interpreted as the level of activation of the pathway.

Activation of the AKT/PI3K, MAPK/ERK1/2, and AMPK signaling pathways was determined by quantifying the total amount of the pathway-specific proteins and their phosphorylated forms. Protein abundances were assessed in protein extracts using the Western Blot technique, as described by Kisielewska et al. [22]. For this analysis, equal amounts of protein extracts, 10 μg per well, were separated on 12.5% SDS-polyacrylamide gels. Proteins were then transferred onto 0.45 µm pore diameter polyvinylidene fluoride (PVDF) membranes (Merck, Darmstadt, Germany) by SEMI-DRY transfer (conditions for two gels: 12V, 5A, 12 min) using an Invitrogen Power Blotter Station (Thermo Fisher Scientific, Waltham, MA, USA). Following that, the membranes were blocked in 5% BSA in tris-buffered saline with 0.1% TWEEN^®^ 20 (TBST; Merck, Darmstadt, Germany) for 1 h. The membranes were then incubated overnight at 4 °C with the appropriate primary antibodies: anti-total-Akt (1:1000; Cat. No. 9272, Cell Signaling Technology, Danvers, MA, USA), anti-phospho-Akt (1:1000; Cat. No. 9271, Cell Signaling Technology, Danvers, MA, USA), anti-total-MAPK (1:1000; Cat. No. 9102, Cell Signaling Technology, Danvers, MA, USA), anti-phospho-MAPK (1:1000; Cat. No. 9101, Cell Signaling Technology, Danvers, MA, USA), anti-total-AMPK (1:1000; Cat. No. 2532, Cell Signaling Technology, Danvers, MA, USA), anti-phospho-AMPK (1:1000; Cat. No. 2531, Cell Signaling Technology, Danvers, MA, USA). The next day, the membranes were incubated with horseradish peroxidase-conjugated goat anti-rabbit secondary antibodies (1:10,000; Cat. No. AP156P, Merck, Darmstadt, Germany) for 1.5 h at RT. Immuno-labeled bands were detected by chemiluminescence using Immobilon Western Chemiluminescent HRP Substrate (Merck, Darmstadt, Germany), according to the manufacturer’s instructions. Membranes were then analyzed and archived using the Azure 280 Imaging System (Azure Biosystems, Dublin, CA, USA). Appropriate-sized bands were identified and protein amounts were quantified by densitometric analysis using Image Studio™ Lite v. 5.2 software (LI-COR Biosciences, Lincoln, NE, USA). The ratio between the amount of phosphorylated pathway-specific protein and its non-phosphorylated (total) form for each sample from the same extraction was interpreted as the level of activation of the pathway.

### 2.7. Statistical Analysis

Statistical analyses were performed using Statistica 13.3 software (StatSoft Inc., Tulsa, OK, USA) and the Python programming language (Python 3.9 Software Foundation, Wilmington, NC, USA) with the Pandas, NumPy, and Statsmodels statistical modules. Prior to the main analyses, all data were tested for assumptions of normality (Shapiro–Wilk test) and homogeneity of variances (Levene’s test). Data were analyzed using one-way, two-way, or multifactorial analysis of variance (ANOVA). For Expt. No. 1, the results of LH and FSH secretion were analyzed using multifactorial ANOVA with four independent variables (main factors): phase, stimulation of cells, vis dose and FK866, and the interaction of these factors. A two-way ANOVA followed by Duncan’s post hoc test was then used to examine the differences in gonadotropins’ secretion levels for each experimental setup and phase of the estrous cycle. Similarly, for Expt. No. 2, the effects of the main factors, i.e., phase, vis dose and FK866, and the interaction of these factors on cell proliferation were determined using multifactorial ANOVA. Then, the differences between groups for each experimental setup and phase of the cycle were determined using two-way ANOVA followed by Duncan’s post hoc test. For Expt. No. 3, to investigate the in vitro effect of VIS (added alone or in combination with FK866) on the apoptosis process, two-way ANOVA followed by Duncan’s post hoc test was used. Subsequently, for Expt. No. 4, a one-way ANOVA was used to determine the time-dependent effect of VIS (at the physiological dose) on the activation of the INSR, AKT/PI3K, MAPK/ERK1/2, and AMPK pathways. Finally, for Expt. No. 5, a multifactorial ANOVA followed by Dunnett’s post hoc test was used to determine the in vitro effect of VIS on LH and FSH secretion levels in the presence of inhibitors of the signaling pathways. This analysis included five independent variables (main factors): VIS and inhibitors, S961, LY294002, U0126, DMPH, and the interactions of these factors. All experimental data are presented as mean ± standard error of the mean (S.E.M.) of experiments performed in five replicates (*n =* 5). Values at *p* < 0.05 were considered statistically significant.

## 3. Results

**Figure 1:**
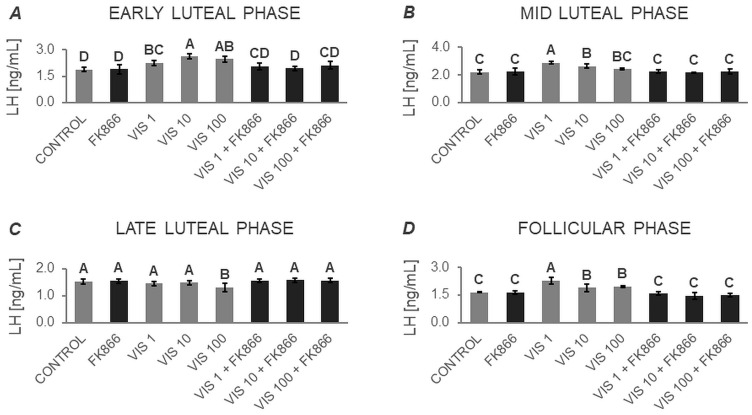
The in vitro effect of visfatin on basal LH secretion by porcine anterior pituitary cells during the estrous cycle. This study was conducted on anterior pituitary glands harvested from pigs (*n* = 5) during the early luteal phase (luteinization phase; days 2–3; (**A**)), mid-luteal phase (the phase of the highest corpus luteum activity throughout the cycle; days 10–12; (**B**)), late luteal phase (luteolysis phase, days 14–16; (**C**)), and the follicular phase (days 17–19; (**D**)). After isolation, anterior pituitary cells were preincubated for 72 h and then incubated for 24 h with visfatin (VIS) at doses of 1, 10, or 100 ng/mL (VIS 1, VIS 10, VIS 100, respectively) or/and FK866 (a selective VIS inhibitor, 10 nM) or serum-free medium alone—CONTROL. The concentration of luteinizing hormone (LH) in the culture media was determined using commercially available ELISA kits. Data were analyzed using a two-way analysis of variance (ANOVA) followed by Duncan’s post hoc test. The results are presented as graphs (mean ± S.E.M.). Bars with different superscripts are significantly different at *p* < 0.05.

**Figure 2:**
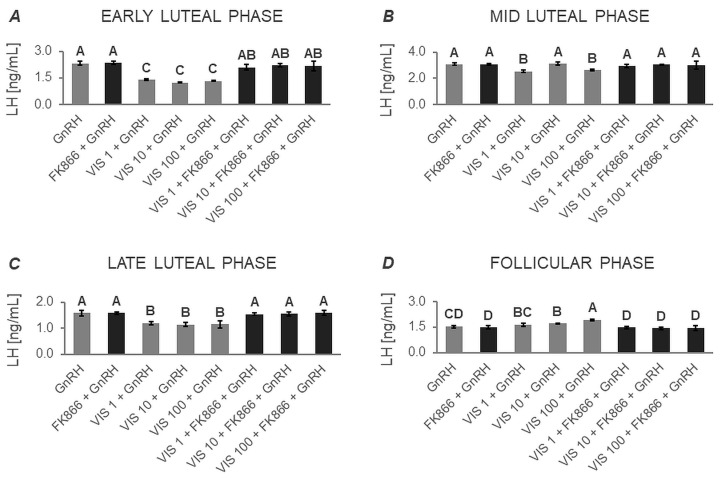
The in vitro effect of visfatin on GnRH-simulated LH secretion by porcine anterior pituitary cells during the estrous cycle This study was conducted on anterior pituitary glands harvested from pigs (*n* = 5) during the early luteal phase (luteinization phase; days 2–3; (**A**)), mid-luteal phase (the phase of the highest corpus luteum activity throughout the cycle; days 10–12; (**B**)), late luteal phase (luteolysis phase, days 14–16; (**C**)), and the follicular phase (days 17–19; (**D**)). After isolation, anterior pituitary cells were preincubated for 72 h and then incubated for 24 h with visfatin (VIS) at doses of 1, 10, or 100 ng/mL (VIS 1, VIS 10, VIS 100, respectively) or/and FK866 (a selective VIS inhibitor, 10 nM) and gonadotropin-releasing hormone (GnRH, 100 ng/mL). The concentration of luteinizing hormone (LH) in the culture media was determined using commercially available ELISA kits. Data were analyzed using a two-way analysis of variance (ANOVA) followed by Duncan’s post hoc test. The results are presented as graphs (mean ± S.E.M.). Bars with different superscripts are significantly different at *p* < 0.05.

**Figure 3:**
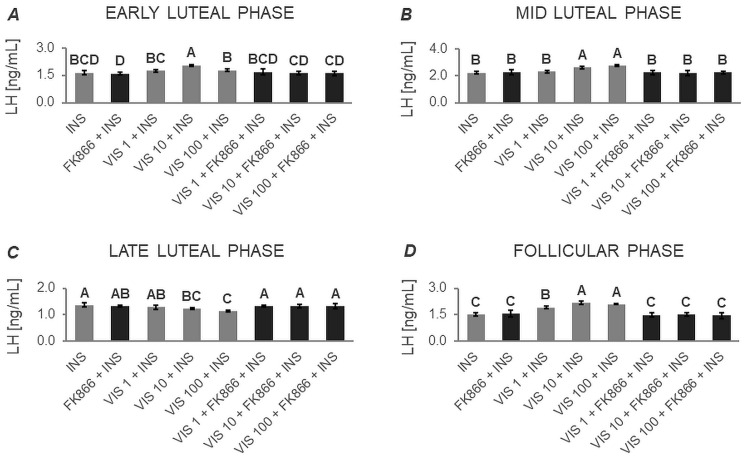
The in vitro effect of visfatin on INS-stimulated LH secretion by porcine anterior pituitary cells during the estrous cycle. This study was conducted on anterior pituitary glands harvested from pigs (*n* = 5) during the early luteal phase (luteinization phase; days 2–3; (**A**)), mid-luteal phase (the phase of the highest corpus luteum activity throughout the cycle; days 10–12; (**B**)), late luteal phase (luteolysis phase, days 14–16; (**C**)), and the follicular phase (days 17–19; (**D**)). After isolation, anterior pituitary cells were preincubated for 72 h and then incubated for 24 h with visfatin (VIS) at doses of 1, 10, or 100 ng/mL (VIS 1, VIS 10, VIS 100, respectively) or/and FK866 (a selective VIS inhibitor, 10 nM) and insulin (INS, 10 ng/mL). The concentration of luteinizing hormone (LH) in the culture media was determined using commercially available ELISA kits. Data were analyzed using a two-way analysis of variance (ANOVA) followed by Duncan’s post hoc test. The results are presented as graphs (mean ± S.E.M.). Bars with different superscripts are significantly different at *p* < 0.05.

**Figure 4:**
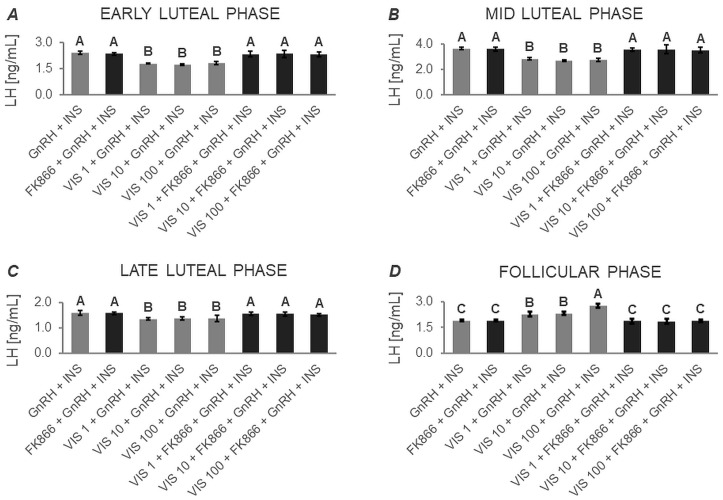
The in vitro effect of visfatin on GnRH + INS-stimulated LH secretion by porcine anterior pituitary cells during the estrous cycle. This study was conducted on anterior pituitary glands harvested from pigs (*n* = 5) during the early luteal phase (luteinization phase; days 2–3; (**A**)), mid-luteal phase (the phase of the highest corpus luteum activity throughout the cycle; days 10–12; (**B**)), late luteal phase (luteolysis phase, days 14–16; (**C**)), and the follicular phase (days 17–19; (**D**)). After isolation, anterior pituitary cells were preincubated for 72 h and then incubated for 24 h with visfatin (VIS) at doses of 1, 10, or 100 ng/mL (VIS 1, VIS 10, VIS 100, respectively) or/and FK866 (a selective VIS inhibitor, 10 nM) and gonadotropin-releasing hormone (GnRH, 100 ng/mL) and insulin (INS, 10 ng/mL). The concentration of luteinizing hormone (LH) in the culture media was determined using commercially available ELISA kits. Data were analyzed using a two-way analysis of variance (ANOVA) followed by Duncan’s post hoc test. The results are presented as graphs (mean ± S.E.M.). Bars with different superscripts are significantly different at *p* < 0.05.

**Figure 5:**
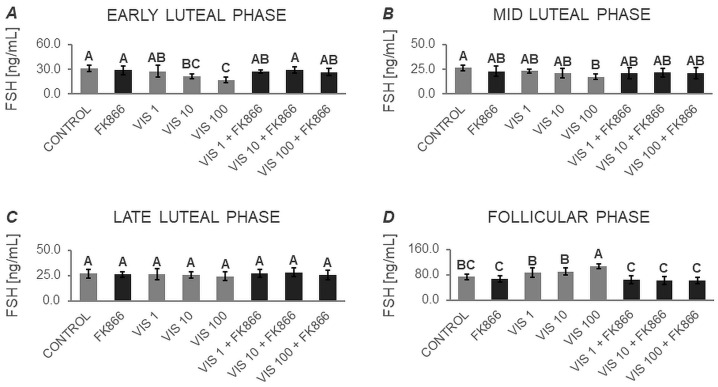
The in vitro effect of visfatin on basal FSH secretion by porcine anterior pituitary cells during the estrous cycle. This study was conducted on anterior pituitary glands harvested from pigs (*n* = 5) during the early luteal phase (luteinization phase; days 2–3; (**A**)), mid-luteal phase (the phase of the highest corpus luteum activity throughout the cycle; days 10–12; (**B**)), late luteal phase (luteolysis phase, days 14–16; (**C**)), and the follicular phase (days 17–19; (**D**)). After isolation, anterior pituitary cells were preincubated for 72 h and then incubated for 24 h with visfatin (VIS) at doses of 1, 10, or 100 ng/mL (VIS 1, VIS 10, VIS 100, respectively) or/and FK866 (a selective VIS inhibitor, 10 nM) or serum-free medium alone—CONTROL. The concentration of follicle-stimulating hormone (FSH) in the culture media was determined using commercially available ELISA kits. Data were analyzed using a two-way analysis of variance (ANOVA) followed by Duncan’s post hoc test. The results are presented as graphs (mean ± S.E.M.). Bars with different superscripts are significantly different at *p* < 0.05.

**Figure 6:**
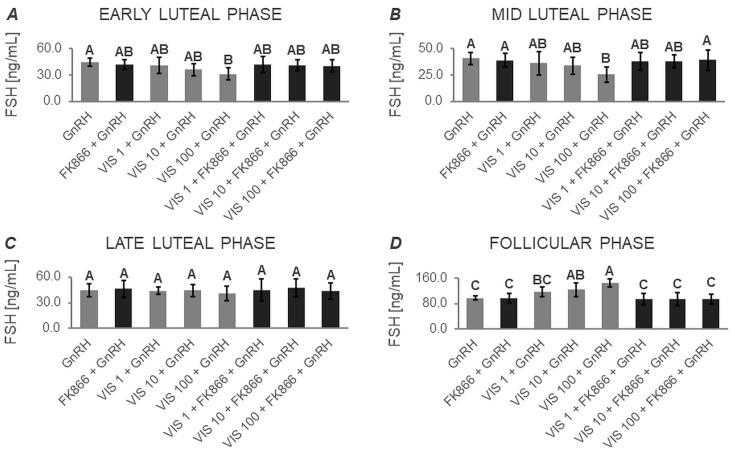
The in vitro effect of visfatin on GnRH-simulated FSH secretion by porcine anterior pituitary cells during the estrous cycle. This study was conducted on anterior pituitary glands harvested from pigs (*n* = 5) during the early luteal phase (luteinization phase; days 2–3; (**A**)), mid-luteal phase (the phase of the highest corpus luteum activity throughout the cycle; days 10–12; (**B**)), late luteal phase (luteolysis phase, days 14–16; (**C**)), and the follicular phase (days 17–19; (**D**)). After isolation, anterior pituitary cells were preincubated for 72 h and then incubated for 24 h with visfatin (VIS) at doses of 1, 10, or 100 ng/mL (VIS 1, VIS 10, VIS 100, respectively) or/and FK866 (a selective VIS inhibitor, 10 nM) and gonadotropin-releasing hormone (GnRH, 100 ng/mL). The concentration of follicle-stimulating hormone (FSH) in the culture media was determined using commercially available ELISA kits. Data were analyzed using a two-way analysis of variance (ANOVA) followed by Duncan’s post hoc test. The results are presented as graphs (mean ± S.E.M.). Bars with different superscripts are significantly different at *p* < 0.05.

**Figure 7:**
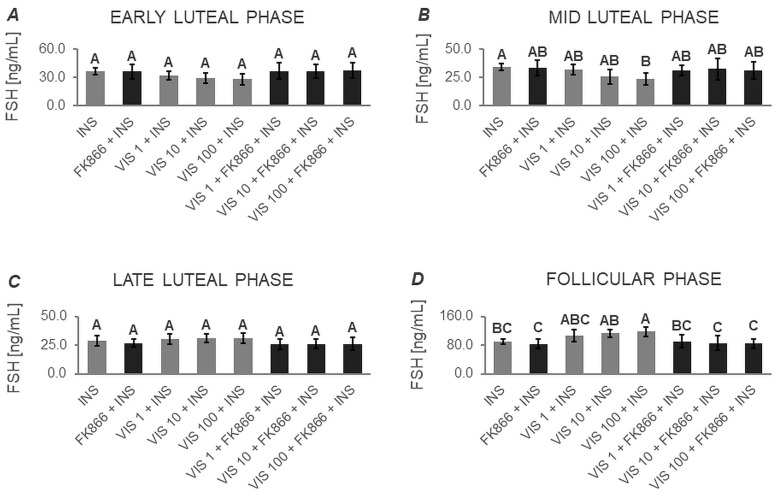
The in vitro effect of visfatin on INS-simulated FSH secretion by porcine anterior pituitary cells during the estrous cycle. This study was conducted on anterior pituitary glands harvested from pigs (*n* = 5) during the early luteal phase (luteinization phase; days 2–3; (**A**)), mid-luteal phase (the phase of the highest corpus luteum activity throughout the cycle; days 10–12; (**B**)), late luteal phase (luteolysis phase, days 14–16; (**C**)), and the follicular phase (days 17–19; (**D**)). After isolation, anterior pituitary cells were preincubated for 72 h and then incubated for 24 h with visfatin (VIS) at doses of 1, 10, or 100 ng/mL (VIS 1, VIS 10, VIS 100, respectively) or/and FK866 (a selective VIS inhibitor, 10 nM) and insulin (INS, 10 ng/mL). The concentration of follicle-stimulating hormone (FSH) in the culture media was determined using commercially available ELISA kits. Data were analyzed using a two-way analysis of variance (ANOVA) followed by Duncan’s post hoc test. The results are presented as graphs (mean ± S.E.M.). Bars with different superscripts are significantly different at *p* < 0.05.

**Figure 8:**
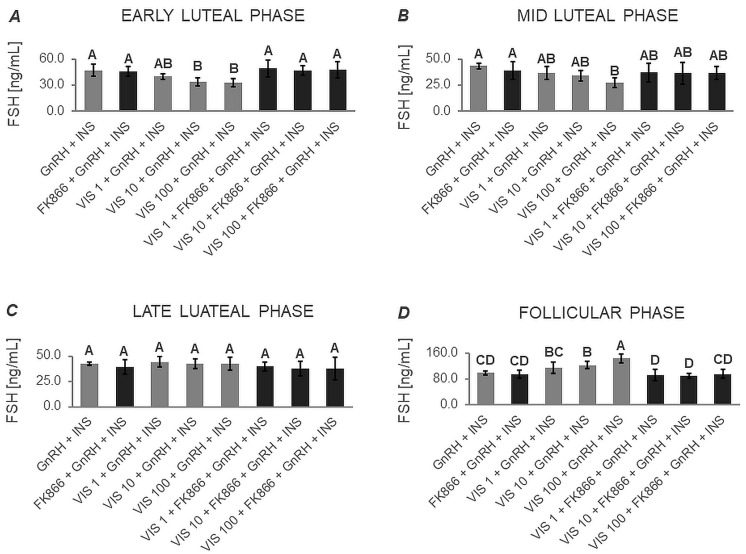
The in vitro effect of visfatin on GnRH + INS-simulated FSH secretion by porcine anterior pituitary cells during the estrous cycle. This study was conducted on anterior pituitary glands harvested from pigs (*n* = 5) during the early luteal phase (luteinization phase; days 2–3; (**A**)), mid-luteal phase (the phase of the highest corpus luteum activity throughout the cycle; days 10–12; (**B**)), late luteal phase (luteolysis phase, days 14–16; (**C**)), and the follicular phase (days 17–19; (**D**)). After isolation, anterior pituitary cells were preincubated for 72 h and then incubated for 24 h with visfatin (VIS) at doses of 1, 10, or 100 ng/mL (VIS 1, VIS 10, VIS 100, respectively) or/and FK866 (a selective VIS inhibitor, 10 nM) and gonadotropin-releasing hormone (GnRH, 100 ng/mL) and insulin (INS, 10 ng/mL). The concentration of follicle-stimulating hormone (FSH) in the culture media was determined using commercially available ELISA kits. Data were analyzed using a two-way analysis of variance (ANOVA) followed by Duncan’s post hoc test. The results are presented as graphs (mean ± S.E.M.). Bars with different superscripts are significantly different at *p* < 0.05.

**Figure 9:**
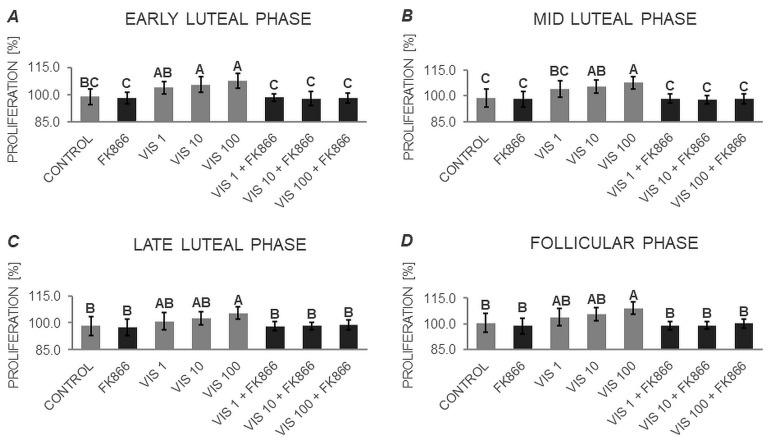
The in vitro effect of visfatin on the proliferation of porcine anterior pituitary cells during the estrus cycle. This study was conducted on anterior pituitary glands harvested from pigs (*n* = 5 per group, 20 in total) during specific phases of the estrus cycle: early luteal phase (luteinization phase; days 2–3; (**A**)), mid-luteal phase (the phase of the highest corpus luteum activity throughout the cycle; days 10–12; (**B**)), late luteal phase (luteolysis phase, days 14–16; (**C**)), and the follicular phase (days 17–19; (**D**)). After isolation, anterior pituitary cells were preincubated for 72 h and then incubated for 24 h with visfatin (VIS) at doses of 1, 10, or 100 ng/mL (VIS 1, VIS 10, VIS 100, respectively) or/and FK866 (a selective VIS inhibitor, 10 nM) or serum-free medium alone—CONTROL. Cell proliferation was assessed immediately after completion of the in vitro culture using an Alamar Blue^®^ assay, according to the manufacturer’s instructions. The obtained results represented the metabolic activity of the cells and indicated the resulting tendency for cell proliferation. Data were analyzed using a two-way analysis of variance (ANOVA) followed by Duncan’s post hoc test. The results are presented as graphs (mean ± S.E.M.). Bars with different superscripts are significantly different at *p* < 0.05.

**Figure 10:**
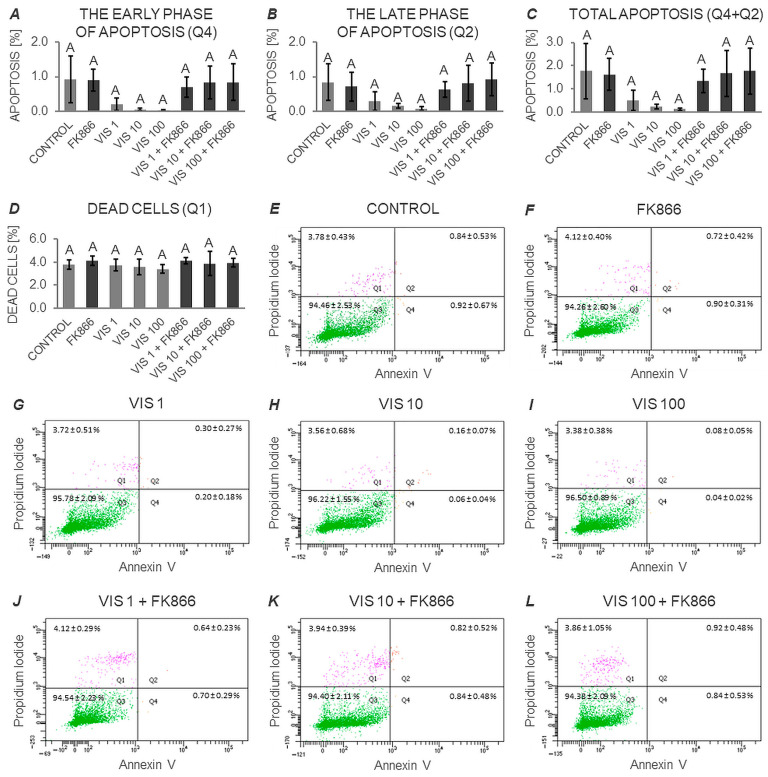
The in vitro effect of visfatin on the apoptosis process in anterior pituitary cells. This study was conducted on anterior pituitary glands harvested from pigs (*n* = 5) during the mid-luteal phase (the phase of the highest corpus luteum activity throughout the cycle; days 10–12). After isolation, anterior pituitary cells were preincubated for 72 h and then incubated for 24 h with visfatin (VIS) at doses of 1, 10, or 100 ng/mL (VIS 1, VIS 10, VIS 100, respectively) or/and FK866 (a selective VIS inhibitor, 10 nM) or serum-free medium alone—CONTROL. Cell apoptosis was assessed immediately after completion of the in vitro culture by dual-color analysis (Annexin V and Propidium Iodide, PI) using a commercially available kit and flow cytometry technique. Both data acquisition and result analysis were performed on 20,000 events. Data were analyzed using a two-way analysis of variance (ANOVA) followed by Duncan’s post hoc test. The results are presented as graphs (mean ± S.E.M.; (**A**–**D**) and scatter plots (**E**–**L**). Bars on graphs with the same superscript are not significantly different at *p* > 0.05. The quadrants of the scatter plot (with the respective numerical values) represent as follows: upper left (Q1)—dead cells (Annexin V negative, PI positive), upper right (Q2)—late apoptotic cells (Annexin V and PI positive), lower left (Q3)—live cells (Annexin V and PI negative), lower right (Q4)—early apoptotic cells (Annexin V positive, PI negative).

**Figure 11:**
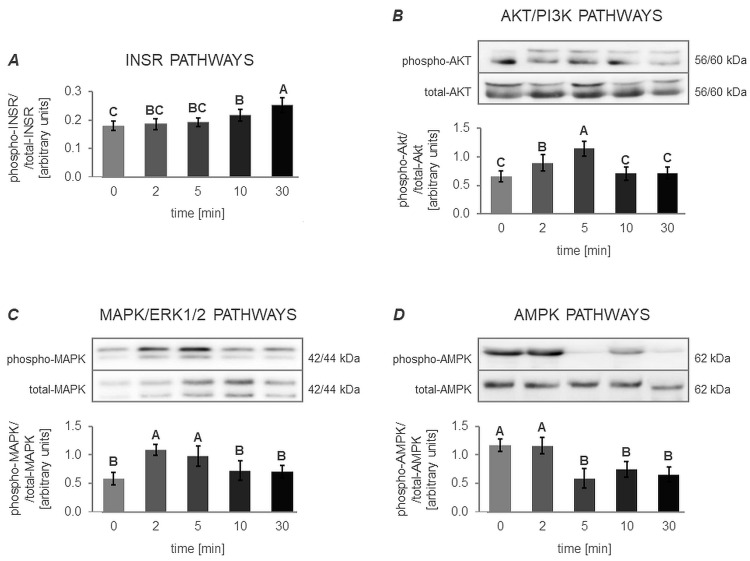
The in vitro effect of visfatin on the activation of the INSR, AKT/PI3K, MAPK/ERK1/2, and AMPK signaling pathways in porcine anterior pituitary cells. This study was conducted on anterior pituitary glands harvested from pigs (*n* = 5) during the mid-luteal phase (the phase of the highest corpus luteum activity throughout the cycle; days 10–12). After isolation, anterior pituitary cells were preincubated for 72 h and then incubated with visfatin (VIS) at a physiological dose (10 ng/mL) for 0, 2, 5, 10, and 30 min. The activation of the insulin receptor (INSR) pathway (**A**) was assessed by quantifying the concentrations of total insulin receptor (total-INSR) and its phosphorylated form (phospho-INSR) in protein extracts using commercially available ELISA kits. In turn, the activation of the protein kinase B/phosphatidylinositol 3-kinase (AKT/PI3K), mitogen-activated protein kinase/extracellular signal-regulated kinase (MAPK/ERK), and adenosine monophosphate-activated protein kinase (AMPK) signaling pathways ((**B**), (**C**), and (**D**), respectively) was determined by quantifying the total amount of the pathway-specific proteins and their phosphorylated forms in protein extracts using the Western Blot technique. Data were analyzed using a one-way analysis of variance (ANOVA) followed by Duncan’s post hoc test. The results are presented as graphs (mean ± S.E.M., for all the signaling pathways) and representative immunoblots (for the AKT/PI3K, MAPK/ERK1/2, and AMPK signaling pathways; uncropped immunoblot images are attached as Supplementary Figures S2–S4). Bars with different superscripts are significantly different at *p* < 0.05.

**Figure 12:**
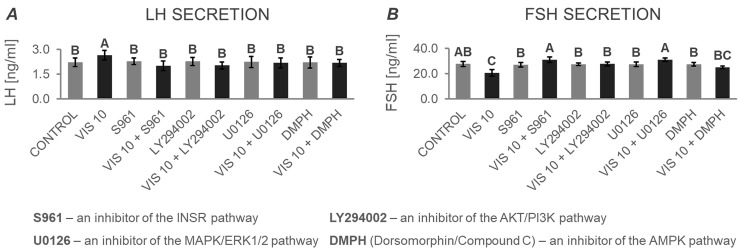
The in vitro effect of visfatin on the secretion of gonadotropins: LH (**A**) and FSH (**B**) by porcine anterior pituitary cells after treatment with inhibitors of the INSR, AKT/PI3K, MAPK/ERK1/2, and AMPK signaling pathways. This study was conducted on anterior pituitary glands harvested from pigs (*n* = 5) during the mid-luteal phase (the phase of the highest corpus luteum activity throughout the cycle; days 10–12). After isolation, anterior pituitary cells were preincubated for 72 h and then incubated for 24 h with visfatin (VIS) at the physiological dose (10 ng/mL, VIS 10), or/and S961—an inhibitor of the insulin receptor pathway (INSR, 1 μM), or/and LY294002—an inhibitor of the protein kinase B/phosphatidylinositol 3-kinase pathway (AKT/PI3K, 20 μM), or/and U0126—an inhibitor of the mitogen-activated protein kinase/extracellular signal-regulated kinase pathway (MAPK/ERK1/2, 10 μM), or/and Dorsomorphin/Compound C (DMPH)—inhibitor of the adenosine monophosphate-activated protein kinase pathway (AMPK, 10 μM) or serum-free medium alone—CONTROL. The concentrations of luteinizing hormone (LH) and follicle-stimulating hormone (FSH) in the culture media were determined using commercially available ELISA kits. Data were analyzed using a multifactorial analysis of variance (ANOVA) followed by Dunnett’s post hoc test. The results are presented as graphs (mean ± S.E.M.). Bars with different superscripts are significantly different at *p* < 0.05.

The F- and *p*-values for the main effects and the interactions of the examined factors are presented in Supplementary Tables S1–S9. In turn, the differences between the groups for each experimental setup and phase of the estrous cycle are presented in Figure 1, Figure 2, Figure 3, Figure 4, Figure 5, Figure 6, Figure 7, Figure 8, Figure 9, Figure 10, Figure 11 and Figure 12. The precise in vitro effect of VIS on gonadotropins’ secretion, cell proliferation, apoptosis, and activation of INSR, AKT/PI3K, MAPK/ERK1/2, and AMPK signaling pathways is described in the following subsections.

### 3.1. The In Vitro Effect of VIS on the Secretion of Gonadotropins (Expt. No. 1)

The multifactorial ANOVAs (Supplementary Tables S1 and S3) showed that LH and FSH secretion levels were influenced by the phase of the estrous cycle, the hormone stimulating the cells, and the dose of VIS (values for the phase, stimulation of cells, and VIS dose, respectively). Furthermore, for LH secretion, the effect of the interactions of almost all the mentioned factors was observed (except vis dose*FK866; Supplementary Table S1), while in the case of FSH secretion, the effect of the interactions phase*stimulation of cells, phase*vis dose, stimulation of cells*vis dose, and phase*stimulation of cells*vis dose was shown (Supplementary Table S3).

#### 3.1.1. Secretion of LH by APc

Based on two-way ANOVA followed by Duncan’s post hoc test (Supplementary Table S2, Figure 1, Figure 2, Figure 3 and Figure 4), VIS, at all the studied doses, was found to enhance the basal secretion of LH during the early luteal and follicular phases (Figure 1A and Figure 1D, respectively). VIS at doses of 1 and 10 ng/mL, also enhanced the basal secretion of LH during the mid-luteal phase (Figure 1B). Conversely, during the late luteal phase, administration of VIS at the highest dose resulted in a decrease in the basal secretion of LH (Figure 1C).

GnRH-stimulated LH secretion was increased in response to VIS at doses of 10 and 100 ng/mL during the follicular phase (Figure 2D). In turn, GnRH-stimulated LH secretion was suppressed by VIS at all the studied doses during the early and late luteal phases (Figure 2A and Figure 2C, respectively). During the mid-luteal phase, VIS at doses of 1 and 100 ng/mL decreased GnRH-stimulated LH secretion (Figure 2B). 

A physiological dose of VIS (10 ng/mL) was found to elevate INS-stimulated LH secretion during the early luteal phase (Figure 3A). VIS at doses of 10 and 100 ng/mL also enhanced INS-stimulated LH secretion during the mid-luteal phase (Figure 3B). Likewise, VIS at all the studied doses increased INS-stimulated LH secretion during the follicular phase (Figure 3D). In contrast, during the late luteal phase, VIS at doses of 10 and 100 ng/mL decreased INS-stimulated LH secretion (Figure 3C).

VIS, at all studied doses, enhanced GnRH + INS-stimulated LH secretion during the follicular phase (Figure 4D). On the other hand, administration of VIS at all studied doses decreased GnRH + INS-stimulated LH secretion during the early, mid-, and late luteal phases (Figure 4A, Figure 4B, and Figure 4C, respectively). 

It should also be noted that no statistically significant changes were observed in basal and GnRH- or/and INS-stimulated LH secretion in the presence of the VIS blocker—FK866 (Figure 1, Figure 2, Figure 3 and Figure 4). The LH secretion levels in the presence of FK866 alone were comparable to those observed in the respective control groups. Similarly, the simultaneous treatment of cells with VIS and FK866 resulted in LH secretion levels comparable to those observed in the respective control groups, effectively negating the modulatory effect of VIS administration alone.

#### 3.1.2. Secretion of FSH by APc

The two-way ANOVA followed by Duncan’s post hoc test (Supplementary Table S4, Figure 5, Figure 6, Figure 7 and Figure 8) demonstrated that the basal secretion of FSH was enhanced by VIS at the highest dose during the follicular phase (Figure 5D). Contrarily, VIS at doses of 10 and 100 ng/mL decreased the basal secretion of FSH during the early luteal phase (Figure 5A). Similarly, VIS at the highest dose suppressed the basal secretion of FSH during the mid-luteal phase (Figure 5B).

GnRH-stimulated FSH secretion was elevated in the presence of VIS at doses of 10 and 100 ng/mL during the follicular phase (Figure 6D). Conversely, GnRH-stimulated FSH secretion was decreased by treatment with the highest VIS dose during the early and mid-luteal phases (Figure 6A and Figure 6B, respectively).

VIS at a dose of 100 ng/mL increased INS-stimulated FSH secretion during the follicular phase (Figure 7D). In turn, the INS-stimulated FSH secretion was decreased by VIS at the highest dose during the mid-luteal phase (Figure 7B).

In response to VIS at doses of 10 and 100 ng/mL, GnRH + INS-stimulated FSH secretion was enhanced during the follicular phase (Figure 8D). On the contrary, VIS at doses of 10 and 100 ng/mL reduced GnRH + INS-stimulated FSH secretion during the early luteal phase (Figure 8A). The highest dose of VIS also suppressed GnRH + INS-stimulated FSH secretion during the mid-luteal phase (Figure 8B).

It is noteworthy that during the late luteal phase, no statistically significant changes were found in basal and GnRH- or/and INS-stimulated FSH secretion (Figure 5C, Figure 6C, Figure 7C and Figure 8C). Similarly, no changes were observed in basal and GnRH- or/and INS-stimulated FSH secretion in the presence of the VIS blocker—FK866. FK866 had no influence on FSH secretion when added by itself, but significantly inhibited VIS effect on the gonadotropin release (Figure 5, Figure 6, Figure 7 and Figure 8).

### 3.2. The In Vitro Effect of VIS on the Proliferation of APc (Expt. No. 2)

Multifactorial ANOVA (Supplementary Table S5) showed that the proliferation of cells was influenced by the phase of the estrus cycle, the dose of VIS (values for the phase and vis dose, respectively), and the interaction of factors phase*vis dose. Further, based on two-way ANOVA followed by Duncan’s post hoc test (Supplementary Table S6, Figure 9), the proliferation of APc was found to be stimulated by VIS at doses of 10 and 100 ng/mL during the early and mid-luteal phases (Figure 9A,B). In addition, cell proliferation was enhanced by the highest dose of VIS during the late luteal and follicular phases (Figure 9C and Figure 9D, respectively). Importantly, no changes in the proliferation of APc were observed in the presence of the VIS inhibitor—FK866. Co-application of VIS and FK866 suppressed the inhibitory effect of VIS to the control level (Figure 9).

### 3.3. The In Vitro Effect of VIS on the Apoptosis Process in Anterior Pituitary Cells (Expt. No. 3)

Performed statistical analysis (two-way ANOVA followed by a Duncan’s post hoc test; Supplementary Table S7 and Figure 10) revealed no significant effect of VIS or/and its inhibitor (FK866) on either the early or late phase of apoptosis of APc (Figure 10A and Figure 10B, respectively), or on the total percentage of apoptotic cells (Figure 10C). Additionally, no significant effect of VIS or/and its inhibitor (FK866) on the number of dead cells was observed (Figure 10D).

### 3.4. The Mechanism of VIS’s Action in Anterior Pituitary Cells

#### 3.4.1. The In Vitro Effect of VIS on the Activation of INSR, AKT/PI3K, MAPK/ERK1/2 and AMPK Signaling Pathway (Expt. No. 4)

One-way ANOVA followed by a Duncan’s post hoc test indicated that VIS affects the activity of the INSR, AKT/PI3K, MAPK/ERK1/2, and AMPK signaling pathways (Figure 11).

In the case of the INSR pathway, an increase in its activity was observed from 10 min onwards, reaching peak activation at 30 min of VIS treatment (Figure 11A). 

For the AKT/PI3K pathway, an increase in activity was observed as early as 2 min, with the pathway reaching its highest level of activation at 5 min of VIS treatment. The activity of this pathway returned to its initial level at 10 min of incubation (Figure 11B).

Regarding the MAPK/ERK1/2 pathway, a significant surge in activity was found at 2 and 5 min of VIS treatment. Similar to the AKT/PI3K pathway, the activity of this pathway returned to baseline at 10 min of incubation (Figure 11C).

Concerning the AMPK pathway, a noticeable decrease in activity was observed at 5, 10, and 30 min of VIS treatment (Figure 11D).

#### 3.4.2. The In Vitro Effect of VIS on the Secretion of Gonadotropins by APc after Treatment with Inhibitors of the INSR, AKT/PI3K, MAPK/ERK1/2, and AMPK Signaling Pathways (Expt. No. 5) 

Based on multifactorial ANOVA followed by Dunnett’s post hoc test, the results obtained in Expt. No. 1 were confirmed—VIS was found to increase basal LH secretion (Figure 12A) and decrease basal FSH secretion (Figure 12B). Furthermore, post hoc testing revealed that the effect of VIS was blocked in the presence of inhibitors of INSR, AKT/PI3K, MAPK/ERK1/2, and AMPK pathways (Figure 12A and Figure 12B, respectively). These findings indicate that VIS may act in porcine APc through all the studied pathways.

## 4. Discussion

To the best of our knowledge, this is the first report that comprehensively demonstrates the role of VIS in the regulation of pituitary function related to female reproduction. To date, only one study has reported the impact of VIS on the secretory function of the pituitary. That study demonstrated that VIS decreased LH secretion in a mouse pituitary cell line in a dose-dependent manner [13]. Our research has further confirmed that VIS modulates gonadotropins’ secretion in a manner that depends not only on the dose of VIS, but also on the phase of the estrous cycle. VIS was found to modulate (enhance or suppress, depending on stimulatory treatment) basal and GnRH- or/and INS-stimulated LH and FSH secretion in porcine APc during the luteal phases. In turn, during the follicular phase, VIS was observed to increase basal and stimulated LH and FSH secretion by these cells. Thus, it is implied by these findings that the influence of VIS on pituitary cells might be attributed to the temporal physiological state/metabolic activity of these cells and/or the hormonal milieu resulting from, among other factors, the phase of the estrous cycle.

While in this study VIS mainly stimulated gonadotropins’ secretion by APc during the follicular phase, our recent study [14] also showed the lowest protein expression of VIS in the AP lobe during the follicular phase and the highest secretion of VIS by APc during this phase throughout the cycle. Moreover, in this study, we also found that VIS secretion by APc during the follicular phase was significantly stimulated by GnRH, INS, LH, and FSH, without any effect of these factors on the protein abundance of VIS. Thus, the obtained results clearly indicated that the observed effect of the studied factors was more related to the extracellular form of VIS (eNAMPT) acting as a hormone, rather than its intracellular form (iNAMPT) acting as an enzyme. It is also noteworthy that the number of GnRH receptors in the pituitary increases during the follicular phase in several species, including pigs [23]. This is mainly due to the elevated plasma GnRH concentration and pulse frequency [24]. In addition, receptors for INS and insulin-like growth factor 1 (IGF-1) are expressed in the APc of various species, with the highest number observed at the end of the cycle, as demonstrated in rats [25,26,27,28]. Consequently, INS can directly enhance the gonadotropin response to GnRH stimulation during the follicular phase. It is also important to note that the follicular phase of the estrous cycle in pigs is characterized by a high concentration of estradiol (E_2_) and a low concentration of progesterone (P_4_) in the peripheral blood. Moreover, P_4_ is known to be a negative regulator of pituitary GnRH receptors [29,30,31,32], whereas E_2_ increases the expression of these receptors [30,33,34,35]. In turn, the number of gonadotrophs (responsible for the production of LH and FSH), which contain the estrogen receptor alpha (ERα) and are thus sensitive to estrogen action, increases in the AP during the follicular phase of the cycle [36]. Estrogens can also increase the abundance of IGF-1 receptors in the APc of several species, including pigs [37]. In addition, a feedback mechanism between IGF-1 and E_2_ has been suggested: estrogens may sensitize APc to IGF-1, and IGF-1 may upregulate E_2_ receptor expression [38]. Therefore, the observed stimulatory effect of VIS on GnRH- and/or INS-stimulated LH and FSH secretion by APc may also be attributed to the increased sensitivity of AP to the actions of GnRH and INS, springing directly from an increased number of their receptors, and indirectly, from a hormonal milieu of the animals resulting from the phase of the estrous cycle.

The literature data have indicated that VIS can stimulate cell proliferation. However, the preponderance of these studies has focused on the impact of VIS on the proliferation of cancer cell lines, with limited information available on its effect on normal primary cells. The stimulatory effect of VIS has been documented in studies concerning endometrial, breast, prostate, liver, colon, and skin cancers [39,40,41,42,43,44]. Moreover, in studies on endometrial and breast cancer cell lines, it has been shown that VIS activated the transition of cells from the G1 to S phase (a critical checkpoint in the cell cycle), and it enhances the rate of DNA synthesis, a crucial step for cell division [39,43]. VIS has also been observed to increase the proliferation of the mouse pancreatic cell line, commonly used in studies on INS synthesis, processing, and secretion [45]. Furthermore, VIS enhanced the proliferation of the mouse gonadotroph cell line [13]. In our study as well, the proliferation of porcine APc was found to be stimulated by VIS during all examined phases of the estrous cycle. Therefore, while VIS has been consistently linked with the stimulation of cell proliferation in a majority of studies, more research is needed to fully understand the exact mechanisms of VIS’s action in cells under physiological conditions.

Although numerous reports on the role of VIS in apoptosis have been published, these studies have provided conflicting results, and the role of VIS in this process remains inconclusive. The stimulatory effect of VIS was found in studies conducted on mouse cells and immune organs [46]. It has been demonstrated that VIS can promote the apoptosis process by decreasing the gene expression of anti-apoptotic Bcl-2 family proteins (*BCL2A1A* and *BCL2L1*) and increasing the gene expression of pro-apoptotic Bcl-2-associated X protein (*BAX*); all the aforementioned factors are associated with the intrinsic pathway of apoptosis. Additionally, it was also found that VIS enhanced the gene expression of the first apoptosis signal (*Fas*)—a receptor of the extrinsic pathway of apoptosis, and caspase 3, which plays a pivotal role in the execution of the apoptotic cell death program. Conversely, the inhibitory effect of VIS was documented in studies on mouse and rat pancreatic cell lines, rat hippocampal cells, and a colon cancer cell line [28,30,31,32]. Furthermore, VIS was observed to exhibit an anti-apoptotic effect by increasing the gene/protein expression of Bcl-2 and decreasing the gene/protein expression of cellular tumor antigen p53 (a key regulator of apoptosis that promotes cell death after extensive DNA damage), cytochrome c (a factor that mediates activation of the intrinsic pathway of apoptosis), and caspase 3 [43,45,47,48,49]. The results obtained in this study suggest that VIS does not trigger cell death. Thus, the findings indicate that the effects of VIS on the apoptosis process may vary based on cell type, cellular environment, and other largely undefined factors. Consequently, a pressing need exists for more comprehensive research to fully understand the molecular mechanisms underlying VIS’s dual role in the apoptotic process.

The AKT/PI3K and MAPK/ERK1/2 signaling pathways are critical kinase cascades that regulate various cellular processes, including cell growth, proliferation, differentiation, survival, and apoptosis [50,51]. It has been demonstrated that VIS increased cell viability and prevented apoptosis through the activation of these pathways in studies on mouse pancreatic, as well as human breast and liver cancer cell lines [45,47,52,53]. Moreover, it is noteworthy that the literature data suggest that VIS binds to INSR at a site different from INS [54]. In turn, the INSR signaling pathway is primarily responsible for regulating glucose metabolism at the cellular level, and upon binding to its ligand, INSR is known to phosphorylate and activate several downstream signaling cascades, including the AKT/PI3K and MAPK/ERK1/2 pathways [55]. In a study on endometrial cancer cell lines [43], it was found that VIS stimulated cell proliferation and inhibited apoptosis through the activation of the INSR, AKT/PI3K, and MAPK/ERK1/2 signaling pathways. In this study, it was confirmed that VIS enhanced the activation of the INSR, AKT/PI3K, and MAPK/ERK1/2 pathways in porcine APc. Furthermore, when these cells were simultaneously treated with VIS and inhibitors of these pathways, the modulatory effect of VIS on gonadotropin secretion was negated. Considering the aforementioned findings and the results we obtained, it is plausible that these signaling pathways may play a significant role in the regulation of VIS action in the porcine pituitary as well.

In this study, it was also demonstrated that VIS inhibited the AMPK phosphorylation in porcine APc. When these cells were simultaneously treated with VIS and an inhibitor of the AMPK pathway, an abolition of the observed impact of VIS on LH and FSH secretion was observed. The study on a rat pancreatic cell line has also shown that VIS increased cell viability and inhibited apoptosis, acting via MAPK/ERK1/2 and AMPK signaling pathways. Interestingly, recent findings suggest that the interplay between VIS and the AMPK signaling pathway might provide a link to processes involved in cell metabolism. Recognized as a cellular energy sensor, AMPK serves as a metabolic checkpoint that regulates various metabolic processes to maintain energy homeostasis and supports cell survival under conditions of energy stress. When cellular energy levels are low, the activation of AMPK is observed to inhibit cell growth and proliferation [56]. In turn, as was mentioned in the Introduction section, VIS has been found to be involved in the biosynthesis of NAD+, a coenzyme essential for energy metabolism, and VIS has been observed to influence the activity of sirtuins, a family of NAD+-dependent enzymes involved in various cellular processes, including metabolism, cell cycle progression, and DNA repair [57]. Consequently, an increased supply of NAD+ might enhance metabolism, potentially promoting cell growth and division. In a recent study, a novel post-translational modification-based signaling route for VIS was unveiled. It has been shown that under biological stress, cells can rapidly orchestrate NAD+ metabolism to support DNA repair through AMPK-mediated phosphorylation of VIS, which reinforces the enzymatic activity of the hormone [58]. Therefore, it is conceivable that VIS may regulate cellular processes, including cell metabolism, acting either directly or indirectly, by influencing NAD+ availability and sirtuin activity or by initiating specific signaling pathways.

This study was conducted using primary cell culture, which accurately represents all cell types found in the anterior lobe of the pituitary gland, including somatotropes, lactotropes, corticotropes, thyrotropes, and gonadotropes. This method is considered the most representative in vitro model of the in vivo state and provides a system that is more physiologically relevant than many immortalized cell lines. Unfortunately, the number of cells that can be procured from a pituitary is often limited. Also, it is not always feasible to provide the cells with all conditions present in the body, given the complex and not fully understood nature of locally acting factors. These can only be fully replicated under in vivo conditions. Nevertheless, this model remains the most effective for examining specific mechanisms or processes at the cellular level, an approach that was adopted in this study. Thus, this research contributes to the existing knowledge about VIS’s role in regulating the porcine pituitary’s function. However, the exact mechanism underlying the action of VIS in the porcine APc has not been fully elucidated and requires further research.

## 5. Conclusions

In summary, the results of this study validate the hypothesis that VIS regulates the functioning of the porcine pituitary, and it was evidenced by VIS’s effect on the secretion of gonadotropins, cell proliferation, and the activation of INSR, AKT/PI3K, MAPK/ERK1/2, and AMPK signaling pathways. In addition, our research showed that VIS affected the mentioned processes in a manner that was dependent on the dose of VIS and/or the phase of the estrous cycle. 

## Figures and Tables

**Table 1:** The experimental setup.

Expt. No.	1	2	3	4	5
Phase of the Estrous Cycle *	the early luteal phase (luteinization phase; days 2–3) the mid-luteal phase (the phase in which the activity of the corpus luteum is the highest throughout the cycle; days 10–12) the late luteal phase (luteolysis phase, days 14–16) the follicular phase (days 17–19)			the mid-luteal phase	
Treatments	CONTROL VIS 1 or VIS 10 or VIS 100 FK866 VIS 1 or VIS 10 or VIS 100 + FK866 GnRH VIS 1 or VIS 10 or VIS 100 + GnRH GnRH + FK866 VIS 1 or VIS 10 or VIS 100 + GnRH + FK866 INS VIS 1 or VIS 10 or VIS 100 + INS INS + FK866 VIS 1 or VIS 10 or VIS 100 + INS + FK866 GnRH + INS VIS 1 or VIS 10 or VIS 100 + GnRH + INS GnRH + INS + FK866 VIS 1 or VIS 10 or VIS 100 + GnRH + INS + FK866	CONTROL + AB^®^ FK866 + AB^®^ VIS 1 or VIS 10 or VIS 100 + AB^®^ VIS 1 or VIS 10 or VIS 100 + FK86 + AB^®^	CONTROL FK866 VIS 1 or VIS 10 or VIS 100 VIS 1 or VIS 10 or VIS 100 + FK866	VIS 10	CONTROL VIS 10 S961 VIS 10 + S961 LY294002 VIS 10 + LY294002 U0126 VIS 10 + U0126 DORSOMORPHIN/ COMPOUND C VIS 10 + DORSOMORPHIN/ COMPOUND C
Incub. Time	24 h	24 h	24 h	0 min 2 min 5 min 10 min 30 min	24 h
Objective of the Expt.	the in vitro effect of VIS on the secretion of LH and FSH by APc in pigs	the in vitro effect of VIS on the proliferation of APc in pigs	the in vitro effect of VIS on the apoptosis of APc in pigs	the in vitro effect of VIS on the activation of INSR, AKT/PI3K, MAPK/ERK1/2, and AMPK signaling pathways in APc in pigs	the in vitro effect of VIS on the secretion of LH and FSH by APc in pigs

EXPT., experiment; *, the phase during which pituitary glands were harvested from pigs; INCUB. TIME, incubation time; APc, anterior pituitary cells; VIS 1, visfatin at a dose of 1 ng/mL; VIS 10, visfatin at a dose of 10 ng/mL; VIS 100, visfatin at a dose of 100 ng/mL; FK866, the specific and selective visfatin inhibitor; GnRH, gonadotropin-releasing hormone; INS, insulin; LH, luteinizing hormone; FSH, follicle-stimulating hormone; AB^®^, Alamar Blue^®^; INSR, insulin receptor; AKT/PI3K, protein kinase B/phosphatidylinositol 3-kinase; MAPK/ERK, mitogen-activated protein kinase/extracellular signal-regulated kinase; AMPK, adenosine monophosphate-activated protein kinase; S961, the inhibitor of INSR signaling pathway; LY294002, the inhibitor of AKT/PI3K signaling pathway; U0126, the inhibitor of MAPK/ERK1/2 signaling pathway; Dorsomorphin/Compound C, the inhibitor of AMPK signaling pathway.

**Table 2:** Specifications of the enzyme-linked immunosorbent assays.

Expt. No.	1 and 5		4	
Target Protein	LH	FSH	phospho-INSR	total-INSR
Catalog Number and Supplier’s Name	Cat. No. EP0105; FineTest Biotech Inc., Nanjing, China	Cat. No. EP0060; FineTest Biotech Inc., Nanjing, China	Cat. No. EIA09483p; Enlibio Biotech Co., Wuhan, China	Cat. No. EIA05929p; Enlibio Biotech Co., Wuhan, China
Elisa Type	competitive ELISA	competitive ELISA	double-antibody sandwich ELISA	double-antibody sandwich ELISA
Detection Range	0.781–50 ng/mL	6.25–400 ng/mL	0.156–10 ng/mL	0.312–20 ng/mL
Sensitivity of the Assay	0.469 ng/mL	<3.75 ng/mL	0.05 ng/mL	0.06 ng/mL
Mean Intra-Assay Coefficient of Variation	2.69 ± 1.60%	3.16 ± 1.76%	1.57 ± 0.81%	1.63 ± 0.83%
Mean Inter-Assay Coefficient of Variation	6.58 ± 1.67%	7.34 ± 1.84%	does not apply	does not apply
R^2^	0.9638	0.9525	0.9998	0.9994

EXPT., experiment; LH, luteinizing hormone; FSH, follicle-stimulating hormone; phospho-INSR, phosphorylated insulin receptor; total-INSR, total insulin receptor; R^2^, the coefficient of determination of the standard curve fitting.

## Data Availability

The data underlying this article will be shared on reasonable request to the corresponding author.

## Supplementary Materials

The following supporting information can be downloaded at: https://www.mdpi.com/article/10.3390/cells12242835/s1, Figure S1: H&E staining of anterior and posterior pituitary; Figure S2: The in vitro effect of visfatin on the activation of the AKT/PI3K signalling pathways in the porcine anterior pituitary cells; Figure S3: The in vitro effect of visfatin on the activation of the MAPK/ERK1/2 signalling pathways in the porcine anterior pituitary cells; Figure S4: The in vitro effect of visfatin on the activation of the AMPK signalling pathways in the porcine anterior pituitary cells; Table S1: The main factors and the interactions of these factors affecting the secretion of LH by the porcine anterior pituitary cells during the estrous cycle (the results of multifactorial analysis of variance); Table S2: The main factors and the interactions of these factors affecting the secretion of LH by the porcine anterior pituitary cells during the estrous cycle (the results of two-way analysis of variance); Table S3: The main factors and the interactions of these factors affecting the secretion of FSH by the porcine anterior pituitary cells during the estrous cycle (the results of multifactorial analysis of variance); Table S4: The main factors and the interactions of these factors affecting the secretion of FSH by the porcine anterior pituitary cells during the estrous cycle (the results of two-way analysis of variance); Table S5: The main factors and the interactions of these factors affecting the proliferation of the porcine anterior pituitary cells during the estrous cycle (the results of multifactorial analysis of variance); Table S6: The main factors and the interactions of these factors affecting the proliferation of the porcine anterior pituitary cells during the estrous cycle (the results of two-way analysis of variance); Table S7: The main factors and the interactions of these factors affecting the apoptosis process in the porcine anterior pituitary cells (the results of two-way analysis of variance); Table S8: The main factors and the interactions of these factors affecting LH secretion by the porcine anterior pituitary cells after treatment with inhibitors of the INSR, AKT/PI3K, MAPK/ERK1/2, and AMPK signaling pathways (the results of multifactorial analysis of variance); Table S9: The main factors and the interactions of these factors affecting FH secretion by the porcine anterior pituitary cells after treatment with inhibitors of the INSR, AKT/PI3K, MAPK/ERK1/2, and AMPK signaling pathways (the results of multifactorial analysis of variance).

## References

[1] Amar A.P., Weiss M.H. (2003). Pituitary Anatomy and Physiology. Neurosurg. Clin. N. Am..

[2] Hong G.K., Payne S.C., Jane J.A. (2016). Anatomy, Physiology, and Laboratory Evaluation of the Pituitary Gland. Otolaryngol. Clin. N. Am..

[3] Estienne A., Bongrani A., Reverchon M., Ramé C., Ducluzeau P.-H., Froment P., Dupont J. (2019). Involvement of Novel Adipokines, Chemerin, Visfatin, Resistin and Apelin in Reproductive Functions in Normal and Pathological Conditions in Humans and Animal Models. Int. J. Mol. Sci..

[4] Wang Y.-Y., Hung A.C., Lo S., Yuan S.-S.F. (2021). Adipocytokines Visfatin and Resistin in Breast Cancer: Clinical Relevance, Biological Mechanisms, and Therapeutic Potential. Cancer Lett..

[5] Rongvaux A., Shea R.J., Mulks M.H., Gigot D., Urbain J., Leo O., Andris F. (2002). Pre-B-Cell Colony-Enhancing Factor, Whose Expression Is up-Regulated in Activated Lymphocytes, Is a Nicotinamide Phosphoribosyltransferase, a Cytosolic Enzyme Involved in NAD Biosynthesis. Eur. J. Immunol..

[6] Dakroub A., Nasser A.S., Younis N., Bhagani H., Al-Dhaheri Y., Pintus G., Eid A.A., El-Yazbi A.F., Eid A.H. (2020). Visfatin: A Possible Role in Cardiovasculo-Metabolic Disorders. Cells.

[7] Lovren F., Pan Y., Shukla P.C., Quan A., Teoh H., Szmitko P.E., Peterson M.D., Gupta M., Al-Omran M., Verma S. (2009). Visfatin Activates ENOS via Akt and MAP Kinases and Improves Endothelial Cell Function and Angiogenesis in Vitro and in Vivo: Translational Implications for Atherosclerosis. Am. J. Physiol.-Endocrinol. Metab..

[8] Lee J.O., Kim N., Lee H.J., Lee Y.W., Kim J.K., Kim H.I., Lee S.K., Kim S.J., Park S.H., Kim H.S. (2015). Visfatin, a Novel Adipokine, Stimulates Glucose Uptake through the Ca2^+^-Dependent AMPK–P38 MAPK Pathway in C2C12 Skeletal Muscle Cells. J. Mol. Endocrinol..

[9] Adeghate E. (2008). Visfatin: Structure, Function and Relation to Diabetes Mellitus and Other Dysfunctions. Curr. Med. Chem..

[10] Van den Bergh R., Morin S., Sass H.J., Grzesiek S., Vekemans M., Florence E., Tran H.T.T., Imiru R.G., Heyndrickx L., Vanham G. (2012). Monocytes Contribute to Differential Immune Pressure on R5 versus X4 HIV through the Adipocytokine Visfatin/NAMPT. PLoS ONE.

[11] Romacho T., Valencia I., Ramos-González M., Vallejo S., López-Esteban M., Lorenzo O., Cannata P., Romero A., San Hipólito-Luengo A., Gómez-Cerezo J.F. (2020). Visfatin/ENampt Induces Endothelial Dysfunction in Vivo: A Role for Toll-Like Receptor 4 and NLRP3 Inflammasome. Sci. Rep..

[12] Dupré S.M., Burt D.W., Talbot R., Downing A., Mouzaki D., Waddington D., Malpaux B., Davis J.R.E., Lincoln G.A., Loudon A.S.I. (2008). Identification of Melatonin-Regulated Genes in the Ovine Pituitary Pars Tuberalis, a Target Site for Seasonal Hormone Control. Endocrinology.

[13] Maillard V., Elis S., Desmarchais A., Hivelin C., Lardic L., Lomet D., Uzbekova S., Monget P., Dupont J. (2017). Visfatin and Resistin in Gonadotroph Cells: Expression, Regulation of LH Secretion and Signalling Pathways. Reprod. Fertil. Dev..

[14] Szymanska K., Zaobidna E., Rytelewska E., Mlyczynska E., Kurowska P., Dobrzyn K., Kiezun M., Kaminska B., Smolinska N., Rak A. (2023). Visfatin in the Porcine Pituitary Gland: Expression and Regulation of Secretion during the Oestrous Cycle and Early Pregnancy. Sci. Rep..

[15] Akins R., Morrissette J. (1968). Gross Ovarian Changes during Estrous Cycle of Swine. Am. J. Vet. Res..

[16] Kaminski T., Kiezun M., Zaobidna E., Dobrzyn K., Wasilewska B., Mlyczynska E., Rytelewska E., Kisielewska K., Gudelska M., Bors K. (2021). Plasma Level and Expression of Visfatin in the Porcine Hypothalamus during the Estrous Cycle and Early Pregnancy. Sci. Rep..

[17] Reverchon M., Cornuau M., Cloix L., Rame C., Guerif F., Royere D., Dupont J. (2013). Visfatin Is Expressed in Human Granulosa Cells: Regulation by Metformin through AMPK/SIRT1 Pathways and Its Role in Steroidogenesis. Mol. Hum. Reprod..

[18] Bogacka I., Siawrys G., Okrasa S., Kaminski T., Przala J. (2002). The Influence of GnRH, Oxytocin and Vasoactive Intestinal Peptide on the Secretion of β-Endorphin and Production of CAMP and CGMP by Porcine Pituitary Cells in Vitro. Anim. Reprod. Sci..

[19] Gavin J.R., Roth J., Neville D.M., De Meyts P., Buell D.N. (1974). Insulin-Dependent Regulation of Insulin Receptor Concentrations: A Direct Demonstration in Cell Culture. Proc. Natl. Acad. Sci. USA.

[20] Elliott A.D., Ustione A., Piston D.W. (2015). Somatostatin and Insulin Mediate Glucose-Inhibited Glucagon Secretion in the Pancreatic α-Cell by Lowering CAMP. Am. J. Physiol.-Endocrinol. Metab..

[21] Zhao Y., Zhang Y., Li J., Zheng N., Xu X., Yang J., Xia G., Zhang M. (2018). MAPK3/1 Participates in the Activation of Primordial Follicles through MTORC1-KITL Signaling. J. Cell. Physiol..

[22] Kisielewska K., Rytelewska E., Gudelska M., Kiezun M., Dobrzyn K., Bogus-Nowakowska K., Kaminska B., Smolinska N., Kaminski T. (2020). Expression of Chemerin Receptors CMKLR1, GPR1 and CCRL2 in the Porcine Pituitary during the Oestrous Cycle and Early Pregnancy and the Effect of Chemerin on MAPK/Erk1/2, Akt and AMPK Signalling Pathways. Theriogenology.

[23] Clapper J., Taylor A. (2011). Components of the Porcine Anterior Pituitary Insulin-like Growth Factor System throughout the Estrous Cycle. Domest. Anim. Endocrinol..

[24] Jayes F.C., Britt J.H., Esbenshade K.L. (1997). Role of Gonadotropin-Releasing Hormone Pulse Frequency in Differential Regulation of Gonadotropins in the Gilt. Biol. Reprod..

[25] Luque R.M., Kineman R.D. (2006). Impact of Obesity on the Growth Hormone Axis: Evidence for a Direct Inhibitory Effect of Hyperinsulinemia on Pituitary Function. Endocrinology.

[26] Navratil A.M., Song H., Hernandez J.B., Cherrington B.D., Santos S.J., Low J.M., Do M.-H.T., Lawson M.A. (2009). Insulin Augments Gonadotropin-Releasing Hormone Induction of Translation in LβT2 Cells. Mol. Cell. Endocrinol..

[27] Buggs C., Weinberg F., Kim E., Wolfe A., Radovick S., Wondisford F. (2006). Insulin Augments GnRH-Stimulated LHβ Gene Expression by Egr-1. Mol. Cell. Endocrinol..

[28] Unger J.W., Lange W. (1997). Insulin Receptors in the Pituitary Gland: Morphological Evidence for Influence on Opioid Peptide-Synthesizing Cells. Cell Tissue Res..

[29] Weiss J.M., Polack S., Treeck O., Diedrich K., Ortmann O. (2006). Regulation of GnRH I Receptor Gene Expression by the GnRH Agonist Triptorelin, Estradiol, and Progesterone in the Gonadotroph-Derived Cell Line AT3-1. Endocrine.

[30] Nett T.M., Turzillo A.M., Baratta M., Rispoli L.A. (2002). Pituitary Effects of Steroid Hormones on Secretion of Follicle-Stimulating Hormone and Luteinizing Hormone. Domest. Anim. Endocrinol..

[31] Cheon M., Park D., Park Y., Kam K., Park S.D., Ryu K. (2000). Progesterone Together with Estrogen Attenuates Homologous Upregulation of Gonadotropin-Releasing Hormone Receptor MRNA in Primary Cultured Rat Pituitary Cells. Endocrine.

[32] Bauer-Dantoin A.C., Weiss J., Jameson J.L. (1995). Roles of Estrogen, Progesterone, and Gonadotropin-Releasing Hormone (GnRH) in the Control of Pituitary GnRH Receptor Gene Expression at the Time of the Preovulatory Gonadotropin Surges. Endocrinology.

[33] Nett T.M., Crowder M.E., Wise M.E. (1984). Role of Estradiol in Inducing an Ovulatory-Like Surge of Luteinizing Hormone in Sheep 1. Biol. Reprod..

[34] Quiñones-Jenab V., Jenab S., Ogawa S., Funabashi T., Weesner G.D., Pfaff D.W. (1996). Estrogen Regulation of Gonadotropin-Releasing Hormone Receptor Messenger RNA in Female Rat Pituitary Tissue. Mol. Brain Res..

[35] Wu J.C., Sealfon S.C., Miller W.L. (1994). Gonadal Hormones and Gonadotropin-Releasing Hormone (GnRH) Alter Messenger Ribonucleic Acid Levels for GnRH Receptors in Sheep. Endocrinology.

[36] Tobin V.A., Pompolo S., Clarke I.J. (2001). The Percentage of Pituitary Gonadotropes with Immunoreactive Oestradiol Receptors Increases in the Follicular Phase of the Ovine Oestrous Cycle. J. Neuroendocr..

[37] Rempel L.A., Clapper J.A. (2002). Administration of Estradiol-17β Increases Anterior Pituitary IGF-I and Relative Amounts of Serum and Anterior Pituitary IGF-Binding Proteins in Barrows. J. Anim. Sci..

[38] Xia Y., Weiss J., Polack S., Diedrich K., Ortmann O. (2001). Interactions of Insulin-like Growth Factor-I, Insulin and Estradiol with GnRH-Stimulated Luteinizing Hormone Release from Female Rat Gonadotrophs. Eur. J. Endocrinol..

[39] Kim J.G., Kim E.O., Jeong B.R., Min Y.J., Park J.W., Kim E.S., Namgoong I.S., Kim Y.I., Lee B.J. (2010). Visfatin Stimulates Proliferation of MCF-7 Human Breast Cancer Cells. Mol. Cells.

[40] Ninomiya S., Shimizu M., Imai K., Takai K., Shiraki M., Hara T., Tsurumi H., Ishizaki S., Moriwaki H. (2011). Possible Role of Visfatin in Hepatoma Progression and the Effects of Branched-Chain Amino Acids on Visfatin-Induced Proliferation in Human Hepatoma Cells. Cancer Prev. Res..

[41] Zhao Q., Li J.Y., Zhang J., Long Y.X., Li Y.J., Guo X.D., Wei M.N., Liu W.J. (2020). Role of Visfatin in Promoting Proliferation and Invasion of Colorectal Cancer Cells by Downregulating SDF-1/CXCR4-Mediated MiR-140-3p Expression. Eur. Rev. Med. Pharmacol. Sci..

[42] Bułdak R.J., Bułdak Ł., Polaniak R., Kukla M., Birkner E., Kubina R., Kabała-Dzik A., Duława-Bułdak A., Żwirska-Korczala K. (2013). Visfatin Affects Redox Adaptative Responses and Proliferation in Me45 Human Malignant Melanoma Cells: An in Vitro Study. Oncol. Rep..

[43] Wang Y., Gao C., Zhang Y., Gao J., Teng F., Tian W., Yang W., Yan Y., Xue F. (2016). Visfatin Stimulates Endometrial Cancer Cell Proliferation via Activation of PI3K/Akt and MAPK/ERK1/2 Signalling Pathways. Gynecol. Oncol..

[44] Ryan S., Kridel S. (2008). Visfatin/Nampt: A Potential Regulator of Prostate Tumor Cell Proliferation, Metabolism, and Survival. Cell Mol. Biol..

[45] Cheng Q., Dong W., Qian L., Wu J., Peng Y. (2011). Visfatin Inhibits Apoptosis of Pancreatic β-Cell Line, MIN6, via the Mitogen-Activated Protein Kinase/Phosphoinositide 3-Kinase Pathway. J. Mol. Endocrinol..

[46] Zhang Z., Xiao K., Wang S., Ansari A.R., Niu X., Yang W., Lu M., Yang Z., Rehman Z.U., Zou W. (2022). Visfatin Is a Multifaceted Molecule That Exerts Regulation Effects on Inflammation and Apoptosis in RAW264.7 Cells and Mice Immune Organs. Front. Immunol..

[47] Xiang R.L., Mei M., Su Y.C., Li L., Wang J.Y., Wu L.L. (2015). Visfatin Protects Rat Pancreatic β-Cells against IFN-γ-Induced Apoptosis through AMPK and ERK1/2 Signaling Pathways. Biomed. Environ. Sci..

[48] Erfani S., Aboutaleb N., Oryan S., Shamsaei N., Khaksari M., Kalalian-Moghaddam H., Nikbakht F. (2015). Visfatin Inhibits Apoptosis and Necrosis of Hippocampus CA3 Cells Following Transient Global Ischemia/Reperfusion in Rats. Int. J. Pept. Res. Ther..

[49] Zhao Q., Long Y., Cheng W., Huang Y., Li J., Li Y., Li X., Guo X., Li Y., Li G. (2022). Visfatin Inhibits Colon Cancer Cell Apoptosis and Decreases Chemosensitivity to 5-FU by Promoting the SDF-1/CXCR4/Akt Axis. Int. J. Oncol..

[50] Chang F., Lee J.T., Navolanic P.M., Steelman L.S., Shelton J.G., Blalock W.L., Franklin R.A., McCubrey J.A. (2003). Involvement of PI3K/Akt Pathway in Cell Cycle Progression, Apoptosis, and Neoplastic Transformation: A Target for Cancer Chemotherapy. Leukemia.

[51] Sun Y., Liu W.-Z., Liu T., Feng X., Yang N., Zhou H.-F. (2015). Signaling Pathway of MAPK/ERK in Cell Proliferation, Differentiation, Migration, Senescence and Apoptosis. J. Recept. Signal Transduct..

[52] Gholinejad Z., Kheiripour N., Nourbakhsh M., Ilbeigi D., Behroozfar K., Hesari Z., Golestani A., Shabani M., Einollahi N. (2017). Extracellular NAMPT/Visfatin Induces Proliferation through ERK1/2 and AKT and Inhibits Apoptosis in Breast Cancer Cells. Peptides.

[53] Miethe C., Torres L., Zamora M., Price R.S. (2021). Inhibition of PI3K/Akt and ERK Signaling Decreases Visfatin-Induced Invasion in Liver Cancer Cells. Horm. Mol. Biol. Clin. Investig..

[54] Saddi-Rosa P., Oliveira C.S., Giuffrida F.M., Reis A.F. (2010). Visfatin, Glucose Metabolism and Vascular Disease: A Review of Evidence. Diabetol. Metab. Syndr..

[55] Boucher J., Kleinridders A., Kahn C.R. (2014). Insulin Receptor Signaling in Normal and Insulin-Resistant States. Cold Spring Harb. Perspect. Biol..

[56] Mihaylova M.M., Shaw R.J. (2011). The AMPK Signalling Pathway Coordinates Cell Growth, Autophagy and Metabolism. Nat. Cell Biol..

[57] Garten A., Petzold S., Körner A., Imai S., Kiess W. (2009). Nampt: Linking NAD Biology, Metabolism and Cancer. Trends Endocrinol. Metab..

[58] Liao X., Huang X., Li X., Qiu X., Li M., Liu R., He T., Tang Q. (2022). AMPK Phosphorylates NAMPT to Regulate NAD ^+^ Homeostasis under Ionizing Radiation. Open Biol..

