# Correction: Szymanska et al. The Effect of Visfatin on the Functioning of the Porcine Pituitary Gland: An In Vitro Study. *Cells* 2023, 12, 2835

**DOI:** 10.3390/cells13201741

**Published:** 2024-10-21

**Authors:** Karolina Szymanska, Edyta Rytelewska, Ewa Zaobidna, Marta Kiezun, Marlena Gudelska, Grzegorz Kopij, Kamil Dobrzyn, Ewa Mlyczynska, Patrycja Kurowska, Barbara Kaminska, Anna Nynca, Nina Smolinska, Agnieszka Rak, Tadeusz Kaminski

**Affiliations:** 1 Department of Animal Anatomy and Physiology, Faculty of Biology and Biotechnology, University of Warmia and Mazury in Olsztyn, 10-719 Olsztyn, Poland; k.szymanska@uwm.edu.pl (K.S.); edyta.rytelewska@uwm.edu.pl (E.R.); marta.kiezun@uwm.edu.pl (M.K.); grzegorz.kopij@uwm.edu.pl (G.K.); barbara.kaminska@uwm.edu.pl (B.K.); anna.nynca@uwm.edu.pl (A.N.); nina.smolinska@uwm.edu.pl (N.S.); 2 Department of Biochemistry, Faculty of Biology and Biotechnology, University of Warmia and Mazury in Olsztyn, 10-719 Olsztyn, Poland; ewa.zaobidna@uwm.edu.pl; 3 Department of Human Histology and Embryology, School of Medicine, Collegium Medicum, University of Warmia and Mazury in Olsztyn, 10-082 Olsztyn, Poland; marlena.gudelska@uwm.edu.pl; 4 Department of Zoology, Faculty of Biology and Biotechnology, University of Warmia and Mazury in Olsztyn, 10-719 Olsztyn, Poland; kamil.dobrzyn@uwm.edu.pl; 5 Laboratory of Physiology and Toxicology of Reproduction, Institute of Zoology and Biomedical Research, Jagiellonian University in Krakow, 30-387 Krakow, Poland; ewa.mlyczynska@uj.edu.pl (E.M.); patrycja.kurowska@uj.edu.pl (P.K.); agnieszka.rak@uj.edu.pl (A.R.); 6 Doctoral School of Exact and Natural Sciences, Jagiellonian University in Krakow, 30-348 Krakow, Poland

## Error in Figure

In the original publication [1], the information provided in Figure 12A (letter designations of the bars) does not match the description of the figure in the text (Section 3.4.2). The corrected version is presented below (Figure 12). The authors state that the scientific conclusions are unaffected. This correction was approved by the Academic Editor. The original publication has also been updated.

## Figures and Tables

**Figure 12:**
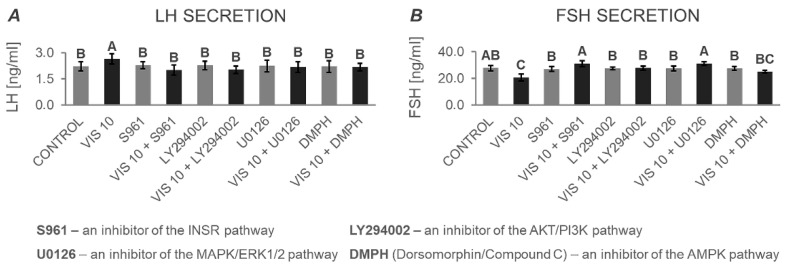
The in vitro effect of visfatin on the secretion of gonadotropins: LH (**A**) and FSH (**B**) by porcine anterior pituitary cells after treatment with inhibitors of the INSR, AKT/PI3K, MAPK/ERK1/2, and AMPK signaling pathways. This study was conducted on anterior pituitary glands harvested from pigs (*n* = 5) during the mid-luteal phase (the phase of the highest corpus luteum activity throughout the cycle; days 10–12). After isolation, anterior pituitary cells were preincubated for 72 h and then incubated for 24 h with visfatin (VIS) at the physiological dose (10 ng/mL, VIS 10), or/and S961—an inhibitor of the insulin receptor pathway (INSR, 1 μM), or/and LY294002—an inhibitor of the protein kinase B/phosphatidylinositol 3-kinase pathway (AKT/PI3K, 20 μM), or/and U0126—an inhibitor of the mitogen-activated protein kinase/extracellular signal-regulated kinase pathway (MAPK/ERK1/2, 10 μM), or/and Dorsomorphin/Compound C (DMPH)—inhibitor of the adenosine monophosphate-activated protein kinase pathway (AMPK, 10 μM) or serum-free medium alone—CONTROL. The concentrations of luteinizing hormone (LH) and follicle-stimulating hormone (FSH) in the culture media were determined using commercially available ELISA kits. Data were analyzed using a multifactorial analysis of variance (ANOVA) followed by Dunnett’s post hoc test. The results are presented as graphs (mean ± S.E.M.). Bars with different superscripts are significantly different at *p* < 0.05.
