# Supplementary material for: The Effect of Visfatin on the Functioning of the Porcine Pituitary Gland: An In Vitro Study
Source: Cells. 2023 Dec 14;12(24):2835. doi: 10.3390/cells12242835 (PMC10742260; doi:10.3390/cells12242835)
Supplement: Supplementary file 1 [file cells-12-02835-s001.zip › cells-2737885-supplementary.pdf]

Supplementary Table S1. The main factors and the interactions of these factors affecting the secretion of LH by the porcine anterior pituitary cells during the estrous cycle (the results of multifactorial analysis of variance)

		FACTORS	F	*p*
Expt. No. 1	LH secretion	PHASE	$F_{3,512} = 2719.20$	0.00000
		STIMULATION OF CELLS	$F_{3,512} = 353.60$	0.00000
		VIS DOSE	$F_{3,512} = 2.70$	0.04245
		FK866	$F_{1,512} = 0.20$	0.62472
		PHASE*STIMULATION OF CELLS	$F_{9,512} = 105.00$	0.00000
		PHASE*VIS DOSE	$F_{9,512} = 12.90$	0.00000
		STIMULATION OF CELLS*VIS DOSE	$F_{9,512} = 23.90$	0.00000
		PHASE*FK866	$F_{3,512} = 138.90$	0.00000
		STIMULATION OF CELLS*FK866	$F_{3,512} = 120.70$	0.00000
		VIS DOSE*FK866	$F_{3,512} = 1.60$	0.18921
		PHASE*STIMULATION OF CELLS*VIS DOSE	$F_{27,512} = 9.70$	0.00000
		PHASE*STIMULATION OF CELLS*FK866	$F_{9,512} = 32.00$	0.00000
		PHASE*VIS DOSE*FK866	$F_{9,512} = 17.30$	0.00000
		STIMULATION OF CELLS*VIS DOSE*FK866	$F_{9,512} = 18.50$	0.00000
		PHASE*STIMULATION OF CELLS*VIS DOSE*FK866	$F_{27,512} = 6.50$	0.00000

Supplementary Table S2. The main factors and the interactions of these factors affecting the secretion of LH by the porcine anterior pituitary cells during the estrous cycle (the results of two-way analysis of variance)

			PHASE OF THE ESTROUS CYCLE	FACTORS	F	*p*
Expt. No. 1	LH	basal secretion	early-luteal phase	VIS	$F_{3,32}$ = 8.67	0.00024
				FK866	$F_{1,32}$ = 22.06	0.00005
				VIS*FK866	$F_{3,32}$ = 5.33	0.00431
			mid-luteal phase	VIS	$F_{3,32}$ = 6.64	0.00129
				FK866	$F_{1,32}$ = 34.48	0.00000
				VIS*FK866	$F_{3,32}$ = 8.09	0.00038
			late-luteal phase	VIS	$F_{3,32}$ = 2.18	0.10974
				FK866	$F_{1,32}$ = 14.53	0.00059
				VIS*FK866	$F_{3,32}$ = 2.74	0.05957
			follicular phase	VIS	$F_{3,32}$ = 7.03	0.00092
				FK866	$F_{1,32}$ = 71.84	0.00000
				VIS*FK866	$F_{3,32}$ = 8.99	0.00018
		GnRH-simulated secretion	early-luteal phase	VIS	$F_{3,32}$ = 39.26	0.00000
				FK866	$F_{1,32}$ = 180.39	0.00000
				VIS*FK866	$F_{3,32}$ = 19.68	0.00000
			mid-luteal phase	VIS	$F_{3,32}$ = 13.12	0.00001
				FK866	$F_{1,32}$ = 14.61	0.00058
				VIS*FK866	$F_{3,32}$ = 6.63	0.00131
			late-luteal phase	VIS	$F_{3,32}$ = 13.87	0.00001
				FK866	$F_{1,32}$ = 95.55	0.00000
				VIS*FK866	$F_{3,32}$ = 11.41	0.00003
			follicular phase	VIS	$F_{3,32}$ = 6.57	0.00138
				FK866	$F_{1,32}$ = 66.49	0.00000
				VIS*FK866	$F_{3,32}$ = 9.20	0.00016
		INS-stimulated secretion	early-luteal phase	VIS	$F_{3,32}$ = 6.95	0.00099
				FK866	$F_{1,32}$ = 27.21	0.00001
				VIS*FK866	$F_{3,32}$ = 6.98	0.00096
			mid-luteal phase	VIS	$F_{3,32}$ = 6.59	0.00135
				FK866	$F_{1,32}$ = 24.86	0.00002
				VIS*FK866	$F_{3,32}$ = 8.87	0.00020
			late-luteal phase	VIS	$F_{3,32}$ = 3.78	0.01980
				FK866	$F_{1,32}$ = 11.74	0.00170
				VIS*FK866	$F_{3,32}$ = 4.56	0.00907
			follicular phase	VIS	$F_{3,32}$ = 10.80	0.00005
				FK866	$F_{1,32}$ = 111.97	0.00000
				VIS*FK866	$F_{3,32}$ = 16.08	0.00000
		GnRH+INS-stimulated secretion	early-luteal phase	VIS	$F_{3,32}$ = 15.51	0.00000
				FK866	$F_{1,32}$ = 97.86	0.00000
				VIS*FK866	$F_{3,32}$ = 14.61	0.00000
			mid-luteal phase	VIS	$F_{3,32}$ = 15.11	0.00000
				FK866	$F_{1,32}$ = 98.02	0.00000
				VIS*FK866	$F_{3,32}$ = 12.54	0.00001
			late-luteal phase	VIS	$F_{3,32}$ = 5.84	0.00268
				FK866	$F_{1,32}$ = 24.30	0.00002
				VIS*FK866	$F_{3,32}$ = 3.63	0.02315
			follicular phase	VIS	$F_{3,32}$ = 22.60	0.00000
				FK866	$F_{1,32}$ = 137.49	0.00000
				VIS*FK866	$F_{3,32}$ = 23.28	0.00000

Supplementary Table S3. The main factors and the interactions of these factors affecting the secretion of FSH by the porcine anterior pituitary cells during the estrous cycle (the results of multifactorial analysis of variance)

		FACTORS	F	*p*
Expt. No. 1	FSH secretion	PHASE	$F_{3,512} = 1630.39$	0.00000
		STIMULATION OF CELLS	$F_{3,512} = 139.91$	0.00000
		VIS DOSE	$F_{3,512} = 34.66$	0.00000
		FK866	$F_{1,512} = 0.29$	0.83584
		PHASE*STIMULATION OF CELLS	$F_{9,512} = 7.93$	0.00000
		PHASE*VIS DOSE	$F_{9,512} = 76.69$	0.00000
		STIMULATION OF CELLS*VIS DOSE	$F_{9,512} = 9.66$	0.00000
		PHASE*FK866	$F_{3,512} = 0.17$	0.99705
		STIMULATION OF CELLS*FK866	$F_{3,512} = 0.13$	0.94042
		VIS DOSE*FK866	$F_{3,512} = 1.10$	0.34832
		PHASE*STIMULATION OF CELLS*VIS DOSE	$F_{27,512} = 10.73$	0.00000
		PHASE*STIMULATION OF CELLS*FK866	$F_{9,512} = 0.98$	0.45427
		PHASE*VIS DOSE*FK866	$F_{9,512} = 0.32$	0.99969
		STIMULATION OF CELLS*VIS DOSE*FK866	$F_{9,512} = 0.06$	0.99995
		PHASE*STIMULATION OF CELLS*VIS DOSE*FK866	$F_{27,512} = 0.21$	1.00000

Supplementary Table S4. The main factors and the interactions of these factors affecting the secretion of FSH by the porcine anterior pituitary cells during the estrous cycle (the results of two-way analysis of variance)

			PHASE OF THE ESTROUS CYCLE	FACTORS	F	*p*
Expt. No. 1	FSH	basal secretion	early-luteal phase	VIS	$F_{3,32} = 5.04$	0.00569
				FK866	$F_{1,32} = 5,82$	0.02176
				VIS*FK866	$F_{3,32} = 3.36$	0.03076
			mid-luteal phase	VIS	$F_{3,32} = 2.19$	0.10853
				FK866	$F_{1,32} = 0.02$	0.88447
				VIS*FK866	$F_{3,32} = 1.12$	0.35613
			late-luteal phase	VIS	$F_{3,32} = 0.37$	0.77751
				FK866	$F_{1,32} = 0.48$	0.49168
				VIS*FK866	$F_{3,32} = 0.25$	0.85770
			follicular phase	VIS	$F_{3,32} = 39.11$	0.00000
				FK866	$F_{1,32} = 2.16$	0.11170
				VIS*FK866	$F_{3,32} = 4.00$	0.01582
		GnRH-simulated secretion	early-luteal phase	VIS	$F_{3,32} = 1.72$	0.18299
				FK866	$F_{1,32} = 1.70$	0.20181
				VIS*FK866	$F_{3,32} = 1.04$	0.38658
			mid-luteal phase	VIS	$F_{3,32} = 1.21$	0.32047
				FK866	$F_{1,32} = 2.31$	0.13801
				VIS*FK866	$F_{3,32} = 1.38$	0.26540
			late-luteal phase	VIS	$F_{3,32} = 0.21$	0.88617
				FK866	$F_{1,32} = 0.46$	0.50350
				VIS*FK866	$F_{3,32} = 0.04$	0.99097
			follicular phase	VIS	$F_{3,32} = 20.77$	0.00007
				FK866	$F_{1,32} = 2.49$	0.07774
				VIS*FK866	$F_{3,32} = 3.02$	0.04396
		INS-stimulated secretion	early-luteal phase	VIS	$F_{3,32} = 4.82$	0.03550
				FK866	$F_{1,32} = 0.44$	0.72388
				VIS*FK866	$F_{3,32} = 0.72$	0.54750
			mid-luteal phase	VIS	$F_{3,32} = 1.60$	0.20766
				FK866	$F_{1,32} = 2.30$	0.13900
				VIS*FK866	$F_{3,32} = 1.11$	0.36042
			late-luteal phase	VIS	$F_{3,32} = 6.88$	0.01327
				FK866	$F_{1,32} = 0,07$	0.97788
				VIS*FK866	$F_{3,32} = 0.19$	0.90072
			follicular phase	VIS	$F_{3,32} = 16.41$	0.00030
				FK866	$F_{1,32} = 1.58$	0.21248
				VIS*FK866	$F_{3,32} = 1.29$	0.29609
		GnRH+INS-stimulated secretion	early-luteal phase	VIS	$F_{3,32} = 15.45$	0.00043
				FK866	$F_{1,32} = 1.86$	0.15617
				VIS*FK866	$F_{3,32} = 2.42$	0.08469
			mid-luteal phase	VIS	$F_{3,32} = 2.41$	0.08388
				FK866	$F_{1,32} = 0.71$	0.40725
				VIS*FK866	$F_{3,32} = 1.37$	0.27029
			late-luteal phase	VIS	$F_{3,32} = 0.15$	0.92881
				FK866	$F_{1,32} = 3.73$	0.06243
				VIS*FK866	$F_{3,32} = 0.02$	0.99665
			follicular phase	VIS	$F_{3,32} = 4.05$	0.01509
				FK866	$F_{1,32} = 34.60$	0.00000
				VIS*FK866	$F_{3,32} = 4.21$	0.01289

Supplementary Table S5. The main factors and the interactions of these factors affecting the proliferation of the porcine anterior pituitary cells during the estrous cycle (the results of multifactorial analysis of variance)

	FACTORS	F	*p*
Expt. No. 2	PHASE	$F_{3,128} = 9.90$	0,00001
	VIS DOSE	$F_{3,128} = 68.60$	0,00000
	FK866	$F_{1,128} = 2.00$	0,11893
	PHASE*VIS DOSE	$F_{9,128} = 6.60$	0,00035
	PHASE*FK866	$F_{3,128} = 0.70$	0,57493
	VIS DOSE*FK866	$F_{3,128} = 0.10$	0,99953
	PHASE*VIS DOSE*FK866	$F_{9,128} = 0.10$	0,99974

Supplementary Table S6. The main factors and the interactions of these factors affecting the proliferation of the porcine anterior pituitary cells during the estrous cycle (the results of two-way analysis of variance)

	**PHASE OF THE ESTROUS CYCLE**	**FACTORS**	**F**	***p***
Expt. No. 2	early-luteal phase	VIS	$F_{3,32} = 22.27$	0.00005
		FK866	$F_{1,32} = 2.26$	0.10066
		VIS*FK866	$F_{3,32} = 2.25$	0.10021
	mid-luteal phase	VIS	$F_{3,32} = 14.37$	0.00063
		FK866	$F_{1,32} = 2.23$	0.10321
		VIS*FK866	$F_{3,32} = 1.66$	0.19497
	late-luteal phase	VIS	$F_{3,32} = 10.54$	0.00274
		FK866	$F_{1,32} = 2.57$	0.07150
		VIS*FK866	$F_{3,32} = 1.19$	0.33033
	follicular phase	VIS	$F_{3,32} = 23.54$	0.00003
		FK866	$F_{1,32} = 2.35$	0.30809
		VIS*FK866	$F_{3,32} = 1.68$	0.19134

Supplementary Table S7. The main factors and the interactions of these factors affecting the apoptosis process in the porcine anterior pituitary cells (the results of two-way analysis of variance)

	APOPTOSIS	FACTORS	F	*p*
Expt. No. 3	the early phase	VIS	$F_{3,32} = 0.28$	0.83850
		FK866	$F_{1,32} = 0.08$	0.78116
		VIS*FK866	$F_{3,32} = 1.63$	0.20123
	the late phase	VIS	$F_{3,32} = 0.10$	0.96014
		FK866	$F_{1,32} = 0.14$	0.70632
		VIS*FK866	$F_{3,32} = 1.38$	0.26741
	total apoptosis	VIS	$F_{3,32} = 0.18$	0.91039
		FK866	$F_{1,32} = 0.11$	0.73936
		VIS*FK866	$F_{3,32} = 1.56$	0.21742
	dead cells	VIS	$F_{3,32} = 0.11$	0.95533
		FK866	$F_{1,32} = 0.80$	0.37754
		VIS*FK866	$F_{3,32} = 0.02$	0.99709

Supplementary Table S8. The main factors and the interactions of these factors affecting LH secretion by the porcine anterior pituitary cells after treatment with inhibitors of the INSR, AKT/PI3K, MAPK/ERK1/2, and AMPK signaling pathways (the results of multifactorial analysis of variance)

		**FACTORS**	**F**	***P***
Expt. No. 5	LH secretion	VIS	$F_{1,39} = 1.27$	0.26687
		S961	$F_{1,39} = 0.61$	0.43938
		S961*VIS	$F_{3,39} = 0.09$	0.76708
		LY294002	$F_{1,39} = 0.44$	0.51135
		LY294002*VIS	$F_{3,39} = 0.07$	0.79725
		U0126	$F_{1,39} = 0.83$	0.48781
		U0126*VIS	$F_{1,39} = 0.78$	0.51453
		DMPH	$F_{3,39} = 0.58$	0.63023
		DMPH*VIS	$F_{3,39} = 0.69$	0.56392

Supplementary Table S9. The main factors and the interactions of these factors affecting FSH secretion by the porcine anterior pituitary cells after treatment with inhibitors of the INSR, AKT/PI3K, MAPK/ERK1/2, and AMPK signaling pathways (the results of multifactorial analysis of variance)

		FACTORS	F	*p*
Expt. No. 5	FSH secretion	VIS	$F_{1,39} = 4.93$	0.03218
		S961	$F_{1,39} = 3.74$	0.06011
		S961*VIS	$F_{3,39} = 5.07$	0.00455
		LY294002	$F_{1,39} = 0.33$	0.57180
		LY294002*VIS	$F_{3,39} = 2.80$	0.05236
		U0126	$F_{1,39} = 0.04$	0.83670
		U0126*VIS	$F_{3,39} = 4.30$	0.04457
		DMPH	$F_{1,39} = 2.46$	0.07677
		DMPH*VIS	$F_{3,39} = 4.93$	0.00522

The purity of the anterior lobe collection is assured by the fact that the anterior and posterior lobes of the porcine pituitary are morphologically distinct tissues, as confirmed by microscopic examination:

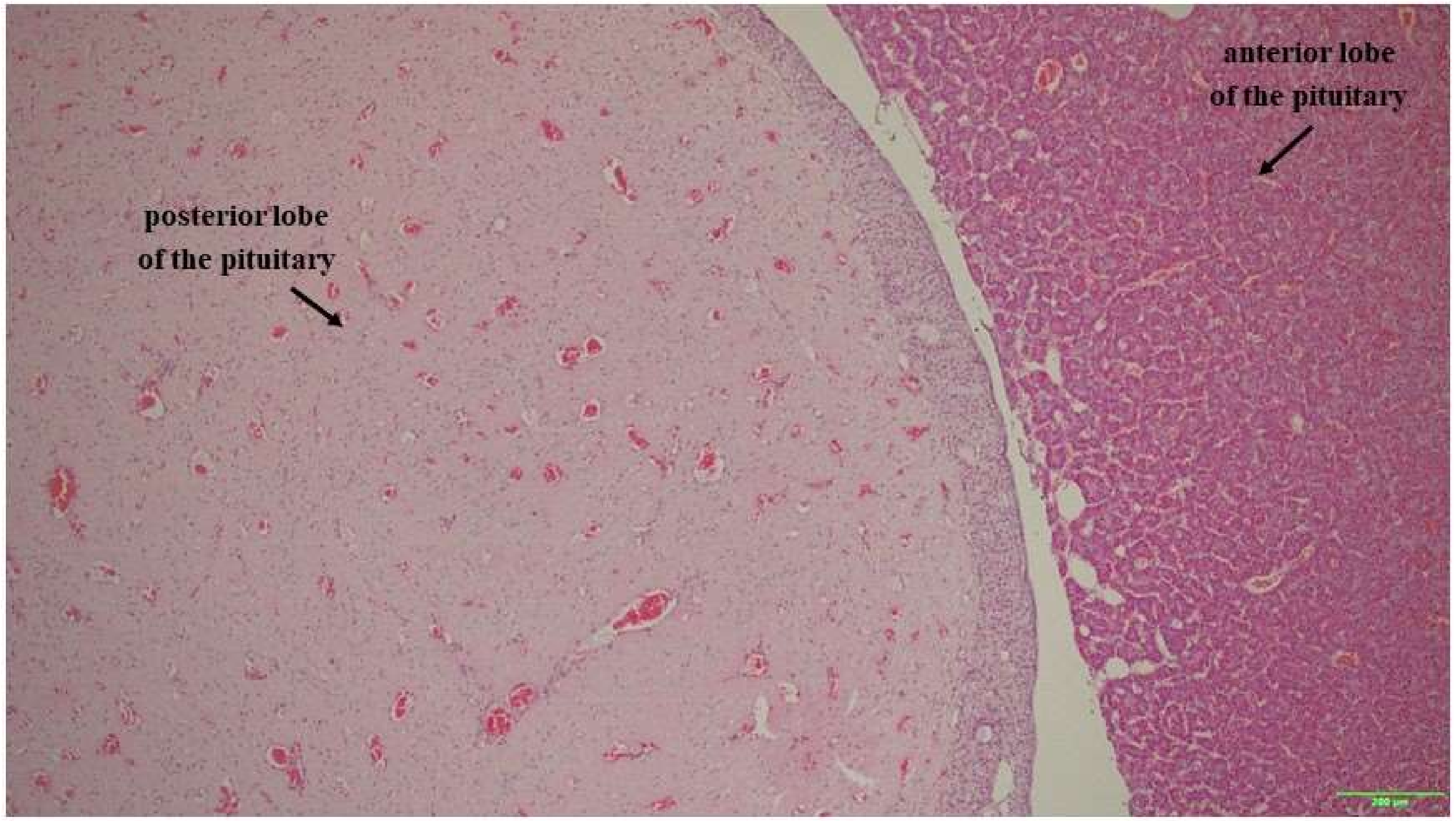

Figure shows the anterior and posterior lobes of the normal pituitary gland of the pig, x4.

Supplementary Figure S1. H&E staining of anterior and posterior pituitary.

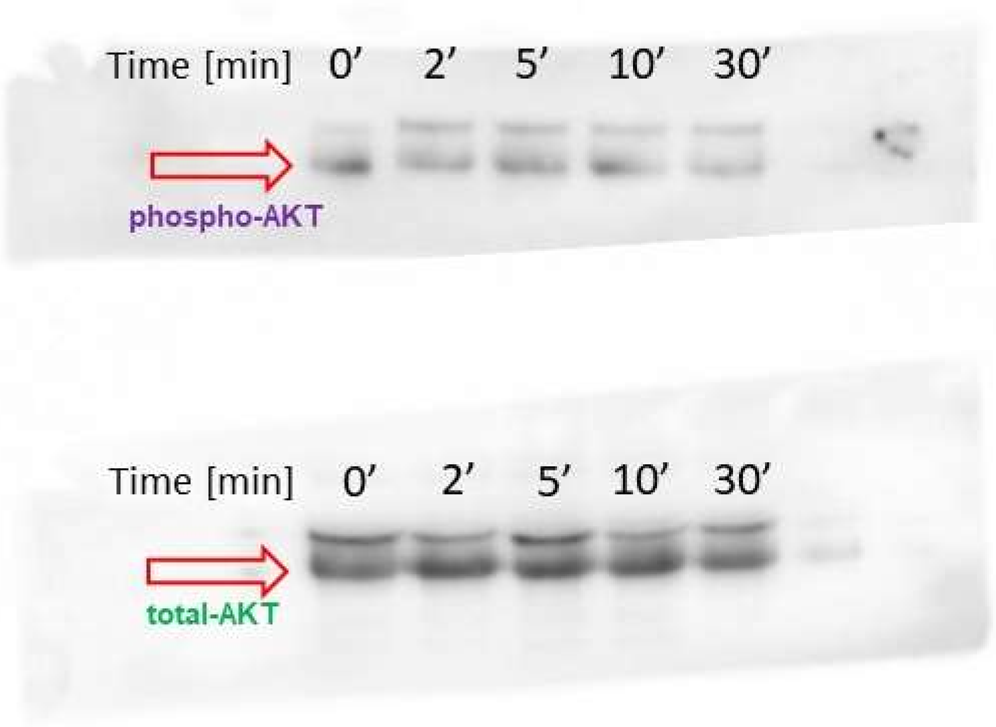

Supplementary Figure S2. The in vitro effect of visfatin on the activation of the AKT/PI3K signalling pathways in the porcine anterior pituitary cells.

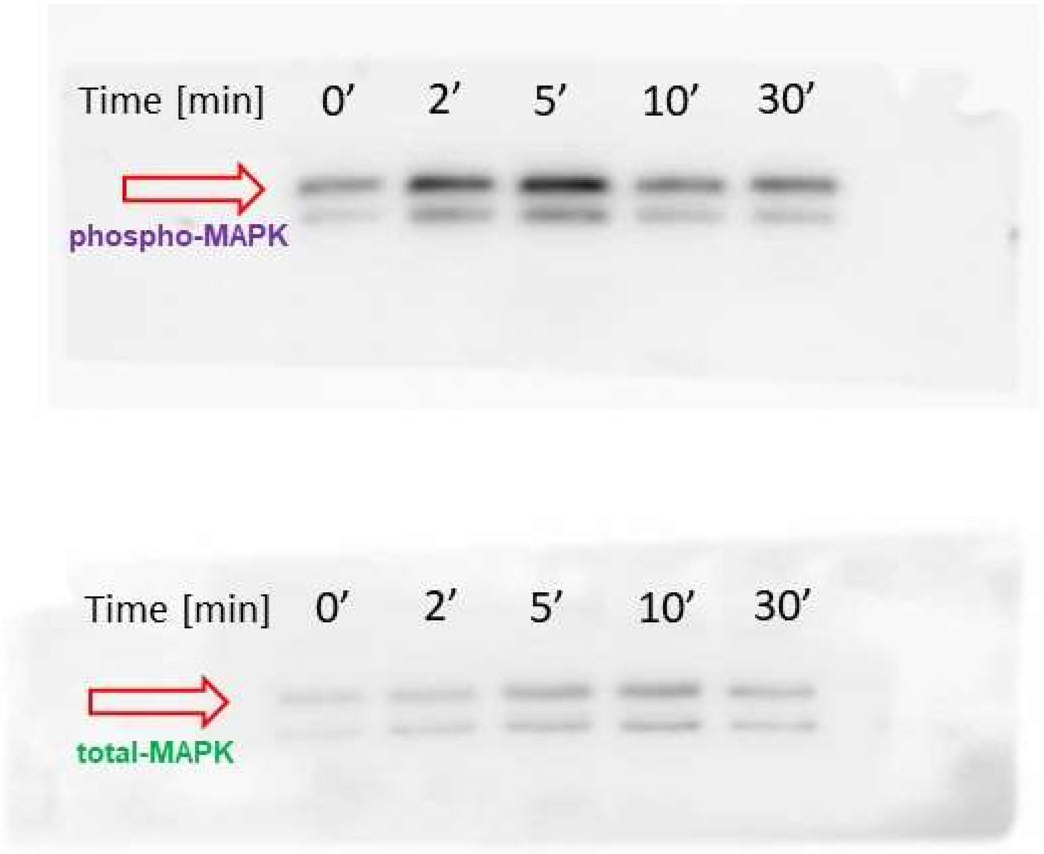

Supplementary Figure S3. The in vitro effect of visfatin on the activation of the MAPK/ERK1/2 signalling pathways in the porcine anterior pituitary cells.

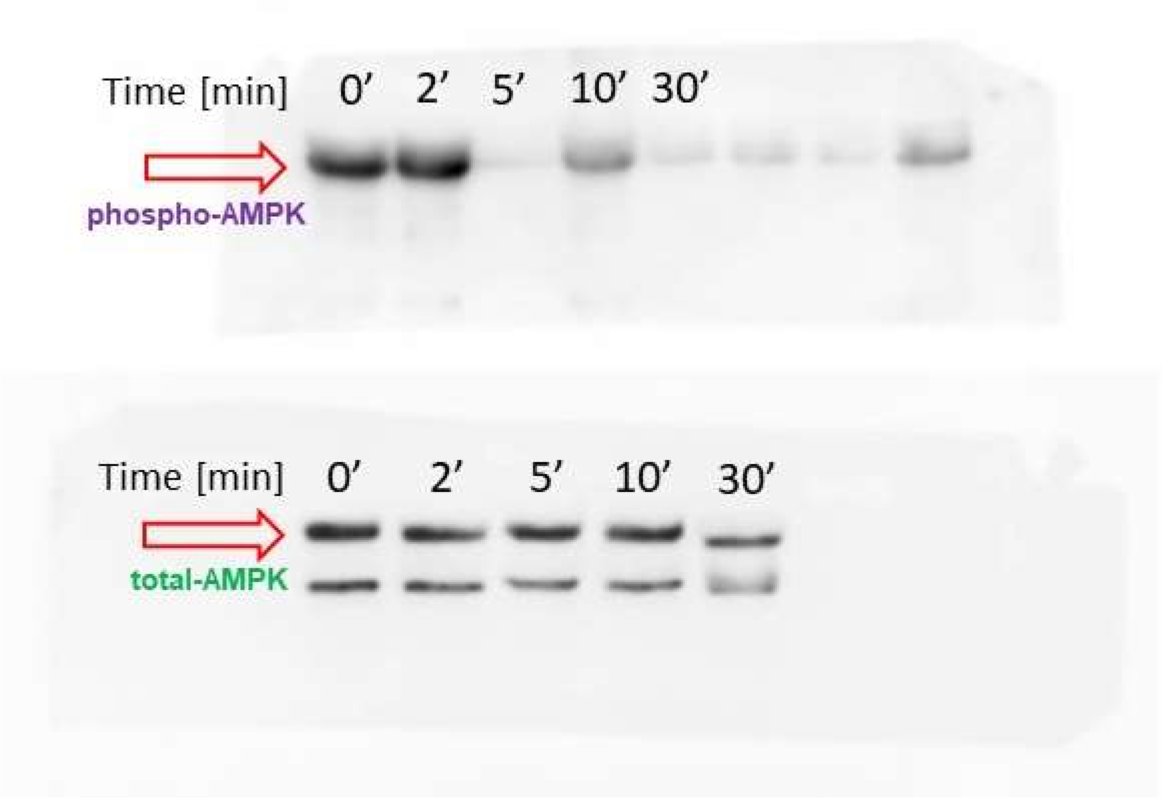

Supplementary Figure S4. The in vitro effect of visfatin on the activation of the AMPK signalling pathways in the porcine anterior pituitary cells.
